# Epidemiology of Non-Melanoma Skin Cancer in Europe over the Last Decade: A Systematic Review

**DOI:** 10.3390/cancers18182970

**Published:** 2026-09-14

**Authors:** Andrzej Ciborek, Hanna Krauss, Zuzanna Chęcińska-Maciejewska, Dariusz Kowalczyk, Jolanta Jaworek

**Affiliations:** 1 Department of Interprofessional Education, Faculty of Medicine and Health Sciences, University of Kalisz, ul. Kaszubska 13, 62-800 Kalisz, Poland; a.ciborek@uniwersytetkaliski.edu.pl; 2 Preventive Research Institute, University of Kalisz, ul. Kaszubska 13, 62-800 Kalisz, Poland; hjk12@poczta.fm; 3 Department of Food and Nutrition, Faculty of Medicine and Health Sciences, University of Kalisz, ul. Kaszubska 13, 62-800 Kalisz, Poland; zuchecinska@gmail.com; 4 Department of Radiology III, Greater Poland Cancer Centre, ul. Kaszubska 12, 62-800 Kalisz, Poland; dariusz.w.kowalczyk@wco.pl; 5 Department of Medicine and Health Care, Faculty of Medicine, University of Applied Sciences in Tarnow, ul. Mickiewicza 8, 33-100 Tarnów, Poland

**Keywords:** non-melanoma skin cancers, basal cell carcinoma, squamous cell carcinoma, epidemiology, incidence, Poland, Europe, UV radiation, cancer registries

## Abstract

Background: Non-melanoma skin cancer (NMSC), principally basal cell carcinoma (BCC) and cutaneous squamous cell carcinoma (cSCC), is incompletely captured by cancer surveillance. Objective: To synthesize European epidemiological evidence, with Poland considered in this context. Methods: PubMed/MEDLINE, Scopus, and Web of Science were searched for publications dated 1 January 2015–31 March 2026, supplemented by registry resources and reference-list searching. Additional eligibility checks during manuscript revision continued until 30 August 2026. The publication-date criterion was distinct from the observation periods of source data. Source-level methodological appraisal informed a descriptive synthesis. Results: The consolidated selection record comprised 198 records, 165 screened records, 61 full-text assessments, 37 full-text exclusions, and 24 included sources. Several registry-based studies reported increasing BCC or cSCC incidence in defined populations. Model-based age-standardized trends varied by geography, sex, age, and observation period. These results do not establish a uniform European increase. Polish estimates were affected by histological aggregation and incomplete ascertainment. Selected pandemic-period studies reported fewer diagnoses or changes in the treated case mix; these findings do not establish a Europe-wide decline in biological incidence or subsequent rebound. Conclusions: NMSC creates a substantial and incompletely measured European burden. Histology-specific registration, explicit multiple-primary counting rules, and improved outpatient capture are priorities for interpreting trends and planning care.

## Simple Summary

Basal cell carcinoma and cutaneous squamous cell carcinoma are common skin cancers that place a substantial demand on healthcare services. This review examines studies and reports published between 1 January 2015 and 31 March 2026 concerning Europe, with Poland considered in this wider context. Several cancer registries report increasing incidence, but estimates derived from statistical models show different patterns across populations and periods. These findings describe different measures and cannot establish one trend for all of Europe. Comparisons are also affected by whether registries record each person or every tumour, distinguish the two cancer types, and capture treatment outside hospitals. Better registration and clearer reporting would improve estimates of disease burden and support prevention and healthcare planning.

## 1. Introduction

### 1.1. Non-Melanoma Skin Cancer in Ageing Populations

Keratinocyte carcinomas, principally basal cell carcinoma (BCC) and cutaneous squamous cell carcinoma (cSCC) account for the majority of non-melanoma skin cancers and generate a substantial cumulative burden because of their high incidence, frequent occurrence of multiple primary lesions, and repeated need for outpatient procedures [1,2,3]. Their apparently low mortality can obscure their importance for health-service capacity, particularly in ageing populations. Epidemiological interpretation is complicated by the use of the aggregate ICD-10 C44 category, heterogeneous rules for counting subsequent tumours, and incomplete reporting of lesions managed outside hospital-based oncology services [3,4,5,6,7]. These limitations affect both temporal trend analysis and international comparisons.

The objective of this systematic review was to critically synthesize European evidence on NMSC incidence, mortality, demographic distribution, registry methodology, and pandemic-period diagnostic disruption reported in publications dated 1 January 2015–31 March 2026. Underlying epidemiological observation periods could begin before 2015. Poland was evaluated within the wider European context. The review addressed three questions: (1) what incidence and mortality patterns were reported within defined populations and data sources; (2) how registration and coding practices affected comparisons; and (3) how Polish findings could be interpreted in relation to the broader European evidence.

### 1.2. Definitions and Characteristics of Non-Melanoma Skin Cancers (Table 1)

The term non-melanoma skin cancer (NMSC) is used in this review primarily for the two major keratinocyte carcinomas, basal cell carcinoma (BCC) and cutaneous squamous cell carcinoma (cSCC). “Keratinocyte carcinoma” is therefore used when evidence specifically concerns BCC and/or cSCC, whereas “NMSC” is retained when it reflects the terminology or aggregated category used by the original source or registry. The following sections summarize the epidemiological characteristics that are most relevant to interpretation of incidence, registration, and disease burden.

**Table 1:** Epidemiologically relevant differences between basal cell carcinoma (BCC) and cutaneous squamous cell carcinoma (cSCC). The table intentionally avoids assigning universal incidence values because published rates depend on population, denominator, standardization, and tumour-counting rules.

Characteristic	BCC	cSCC
ICD-10 coding	C44; topography-based and does not distinguish histology	C44; topography-based and does not distinguish histology
Approximate proportion of keratinocyte carcinomas	~70–80%	~20–30%
Sex distribution	Generally, more frequent in men overall; female predominance may occur in younger age groups in some populations	Usually more frequent in men
Typical anatomical distribution	Predominantly sun-exposed skin, particularly the head and neck	Predominantly chronically sun-exposed skin, especially the head/neck and dorsal upper extremities; distribution may differ in severely immunosuppressed patients
Major epidemiological risk factors	Cumulative UV exposure; age; fair-skin phenotype; immunosuppression	Cumulative UV exposure; age; immunosuppression, particularly solid-organ transplantation and hematologic disease; chronic inflammatory or scarred skin
Precursor lesion	No obligatory precursor lesion	Actinic keratosis is an important precursor lesion
Metastatic potential	Very low in conventional BCC	Higher than BCC; clinically relevant particularly in high-risk cSCC
Epidemiological registration issue	Incidence may be underestimated when subsequent primary tumours and outpatient-treated lesions are not captured	Histology-specific ascertainment is required; invasive and in situ lesions and subsequent primary tumours should be distinguished

### 1.3. Characteristics of Basal Cell Carcinoma and Cutaneous Squamous Cell Carcinoma

BCC is the most common skin cancer [1,6,7] and accounts for approximately 70–80% of NMSCs in many series, whereas cSCC accounts for a smaller but clinically important proportion. Cumulative ultraviolet (UV) exposure is a major determinant of both tumour types. cSCC is additionally associated with chronic immunosuppression, including solid-organ transplantation and hematologic or other immunosuppressive conditions, as well as chronic inflammatory or ulcerative lesions [1,8]. Most cSCCs arise on chronically sun-exposed skin, particularly the head and neck and the dorsal upper extremities; lesions at less sun-exposed sites may be relatively more prominent in severely immunosuppressed populations [8]. BCC also shows a strong predilection for sun-exposed sites, especially the head and neck [1,9].

Sex distributions vary by age, population, and tumour subtype. BCC is relatively more evenly distributed by sex, although sex-specific rates vary between populations and age groups, whereas cSCC generally shows a clearer male predominance in European studies [9,10,11,12,13,14,15]. Incidence of both BCC and cSCC increases steeply with age and cumulative UV exposure; the rise in cSCC at older ages is additionally relevant in the context of immunosenescence and increasing exposure to immunosuppressive treatment [10,11,12,13,14,15,16,17,18]. Reported mean or median ages at diagnosis are population-specific and should not be interpreted as universal thresholds.

The reported incidence of BCC and cSCC is strongly influenced by UV exposure, latitude, population age structure, skin phenotype, and registry methodology. Published European estimates vary widely and are not directly comparable when denominators, standard populations, histological definitions, and rules for counting multiple tumours differ [3,7,18]. Accordingly, numerical rates in this review are presented with their original denominators and methodological context rather than converted into a single pooled European estimate. The ratio of BCC to cSCC is commonly several-fold higher for BCC, while the relative contribution of cSCC increases in older and immunosuppressed populations [1,9].

### 1.4. ICD-10 C44 Classification and Registration Issues

ICD-10 C44 is a topography-based category for malignant neoplasms of skin at specified and unspecified sites; it does not, by itself, distinguish BCC from cSCC histology. Consequently, registry outputs based only on C44 may combine biologically and clinically distinct keratinocyte carcinomas and may also differ in whether in situ disease is captured. European registries further vary in whether they record every tumour, only the first tumour per patient, selected invasive lesions, or cases linked to histopathology. These differences can materially alter reported incidence and make direct cross-country comparisons unreliable [4,6,7].

A local recurrence and a new primary tumour represent different outcomes. First-tumour-only registration can underestimate the burden of subsequent primary lesions and related procedures [5,7,15]. Person-based incidence and tumour-based workload are therefore not interchangeable. Histology-specific coding is also relevant to analysis of age, sex and mortality. Lesions treated without histological confirmation may be incompletely captured by pathology-linked registries.

In Poland, the registered NMSC burden is likely underestimated because a substantial proportion of low-risk lesions are managed outside hospital-based oncology pathways, BCC and cSCC may be aggregated under ICD-10 C44, and subsequent primary tumours may not be captured consistently. These limitations hinder accurate estimation of the true tumour burden and international comparison [1,4,5,7,19,20].

## 2. Materials and Methods

### 2.1. Reporting Guideline, Review Design, and Registration

This systematic review is reported with reference to the PRISMA 2020 statement [21]. A descriptive synthesis was used because the included sources differed in registry design, case definitions, tumour-counting rules, population coverage, rate standardization, and outcome reporting. No meta-analysis was undertaken. The PRISMA checklist is provided as Supplementary Table S1.

The review was not prospectively registered, and no publicly accessible protocol was prepared.

### 2.2. Eligibility Criteria

Eligibility was assessed using the following domains:

- Population/setting: general-population or clinically defined populations in Poland or other European countries. Studies conducted exclusively outside Europe were excluded unless epidemiological results for a European population were reported separately and were extractable.
- Condition: NMSC/keratinocyte carcinoma, BCC, or cSCC. Studies focused exclusively on melanoma were excluded.
- Outcomes: quantitative epidemiological indicators, including case counts, crude or age-standardized incidence, mortality, temporal trends, annual percentage change, demographic distribution, registry completeness, or pandemic-related changes in diagnosis.
- Study/report types: population-based epidemiological studies, cancer-registry analyses, national or international epidemiological reports, and systematic reviews containing extractable epidemiological data.

Publication period and language: publications dated 1 January 2015–31 March 2026, in English or Polish. This restriction applied to publication date, not to the underlying epidemiological observation period. Eligible publications could therefore analyse data collected before 2015.

- Exclusions: studies conducted exclusively outside Europe without separately extractable European data; case reports; small case series; experimental or molecular-only studies without epidemiological outcomes; melanoma-only studies; and publications without eligible quantitative epidemiological indicators. The outcome criterion was operationalized to exclude reports in which NMSC appeared only as an incidental etiologic, pharmacovigilance, treatment-safety, or pan-cancer endpoint without a separately interpretable NMSC measure of burden, incidence/prevalence, temporal trend, mortality, registration/ascertainment, or pandemic-related diagnostic/care disruption. Narrative clinical reviews, guidelines, and study protocols without outcome data were not treated as included epidemiological sources.

### 2.3. Information Sources

PubMed/MEDLINE, Scopus, and Web of Science Core Collection were searched. Supplementary sources comprised the Polish National Cancer Registry and the “Malignant Tumours in Poland” reports, the European Cancer Information System (ECIS), the Global Cancer Observatory (GLOBOCAN/IARC) [22], and reference lists of eligible publications and relevant reviews. The initial searches were dated 31 March 2026. Additional literature checks during manuscript revision continued until 30 August 2026; reports identified in this process were assessed against the review eligibility criteria. These checks were supplementary to the initial search and are not presented as a complete rerun of all three databases.

Supplementary Methods S1 provides the reported database-specific strategies, limits and retrieval counts. Supplementary-source searching was targeted and did not comprise an equally exhaustive search of every European national registry website. Differences in the availability and intensity of grey-literature retrieval were considered a potential source of geographical availability bias.

### 2.4. Search Strategy

The search combined controlled vocabulary, where available, with free-text terms relating to NMSC, BCC, cSCC, epidemiological outcomes, and European geography. Syntax was adapted to each database. Supplementary Methods S1 presents the reported search strings, field tags, Boolean operators, date and language limits, and supplementary-source procedures. Limits described separately from a query are distinguished from restrictions encoded in the search string.

Eligibility was restricted to English- or Polish-language publications dated 1 January 2015–31 March 2026. This criterion concerned publication date, not the years of epidemiological observation, and was retained for the additional checks during revision. Records identified after the initial search were therefore not automatically eligible. The PubMed strategy used an exact publication-date range; Scopus and Web of Science used year-level limits spanning 2015–2026. Eligibility assessment applied the review publication-date criterion to records retrieved within these broader year-level limits.

### 2.5. Selection Process

Following identification and deduplication, study selection comprised title/abstract screening and full-text eligibility assessment. All five authors contributed to these assessments collaboratively. Supplementary Table S3 records excluded full-text reports and one principal reason for exclusion for each report. Publications used only for clinical, prevention, or methodological context were distinguished from sources included in the epidemiological synthesis. No automated screening tools were used.

### 2.6. Data Collection Process and Data Items

Data extraction was undertaken collaboratively by the five authors. Extracted items comprised country or region, publication date and observation period, population and setting, data source and study design, tumour subtype, case definition, ascertainment, handling of first and subsequent primary tumours and in situ lesions, case counts, crude and age-standardized incidence and mortality rates, reported trend measures, age and sex distributions, and indicators of reporting completeness. Pandemic-related diagnostic and care outcomes were extracted separately from incidence outcomes. Missing values were not imputed.

The evidence matrix distinguishes population-based registry data, model-based estimates, administrative claims, and clinical or occupational samples. Rates are interpreted with their denominator, standard population where reported, geographic coverage, sex, age group, and observation period. Annual percentage change (APC) estimated annual percentage change (EAPC), and average annual percentage change (AAPC) retain the definitions and periods used in the source; they are not interchangeable with percentage differences between two counts.

Potential overlap was considered by comparing country, registry or data source, study years, population coverage, and case definition. Sources providing complementary information were retained but were not treated as statistically independent observations or pooled. Supplementary Table S2 presents the source-level extraction matrix. The manuscript reference number is the shared source identifier linking Supplementary Tables S2 and S2B.

### 2.7. Descriptive Source-Level Methodological Appraisal

The five authors jointly undertook a structured descriptive source-level methodological appraisal, which was used to support the interpretation of the heterogeneous evidence base. The nine domains were population coverage, case ascertainment, histopathological confirmation, reporting completeness, distinction between BCC and cSCC, handling of multiple primary tumours, rate standardization, duration of observation, and transparency of statistical methods. Supplementary Table S2B provides domain-level judgments, an overall methodological concern judgment, and a rationale for each of the 24 included sources.

Judgments were expressed as low concern, some concern, or high concern in relation to the intended use of the source in this descriptive synthesis. The framework was not a validated risk-of-bias tool, did not produce a numerical score, and did not constitute a formal certainty-of-evidence grading system. Overall methodological concern reflected the pattern of limitations and the intended interpretation rather than an arithmetic sum or a worst-domain rule. No source was excluded solely because of its appraisal. A low overall judgment does not imply suitability for every comparison; for example, reliable first-diagnosis incidence does not quantify the workload arising from subsequent tumours.

### 2.8. Effect Measures and Synthesis Methods

The synthesis considered case counts, crude rates, age-standardized incidence rates (ASIRs), age-standardized mortality rates (ASMRs), and source-reported APC, EAPC, and AAPC estimates. Findings were grouped by source type, tumour subtype, geography, sex, age, and observation period. Registry-based findings and model-based estimates were described separately, with a further distinction between absolute counts, crude rates, and age-standardized rates within each source type. Registry-based rates can also be age-standardized; age standardization does not identify a result as model-based.

A descriptive synthesis was used because differences in ascertainment, coverage, tumour-counting rules, standard populations, and outcome definitions precluded a defensible pooled estimate. Counts, first-person incidence, tumour-based rates, and rates standardized to different populations were not treated as directly comparable. Author-derived annual changes based on only two time points were not used as substitutes for source-reported regression-based trend estimates. Cross-sectional burden, age gradients, and projections were not treated as observed temporal incidence trends. No formal sensitivity analyses were undertaken.

### 2.9. Reporting Bias and Certainty of Evidence

No formal statistical assessment of reporting bias or quantitative certainty grading was undertaken. The heterogeneous evidence base did not provide a common set of comparable effect estimates for a meaningful small-study-effects analysis. Potential language bias, geographical availability bias, selective accessibility of registry reports, and incomplete ascertainment were considered qualitatively.

GRADE was not applied. Interpretation was informed by source-level methodological appraisal, population coverage, transparency, consistency within comparable outcomes, and susceptibility to changes in ascertainment. This qualitative interpretation was not presented as a validated certainty assessment. Agreement between overlapping reports was not regarded as independent corroboration, and no single confidence judgment was assigned to a Europe-wide incidence trend.

## 3. Results

### 3.1. Study Selection

**Figure 1:**
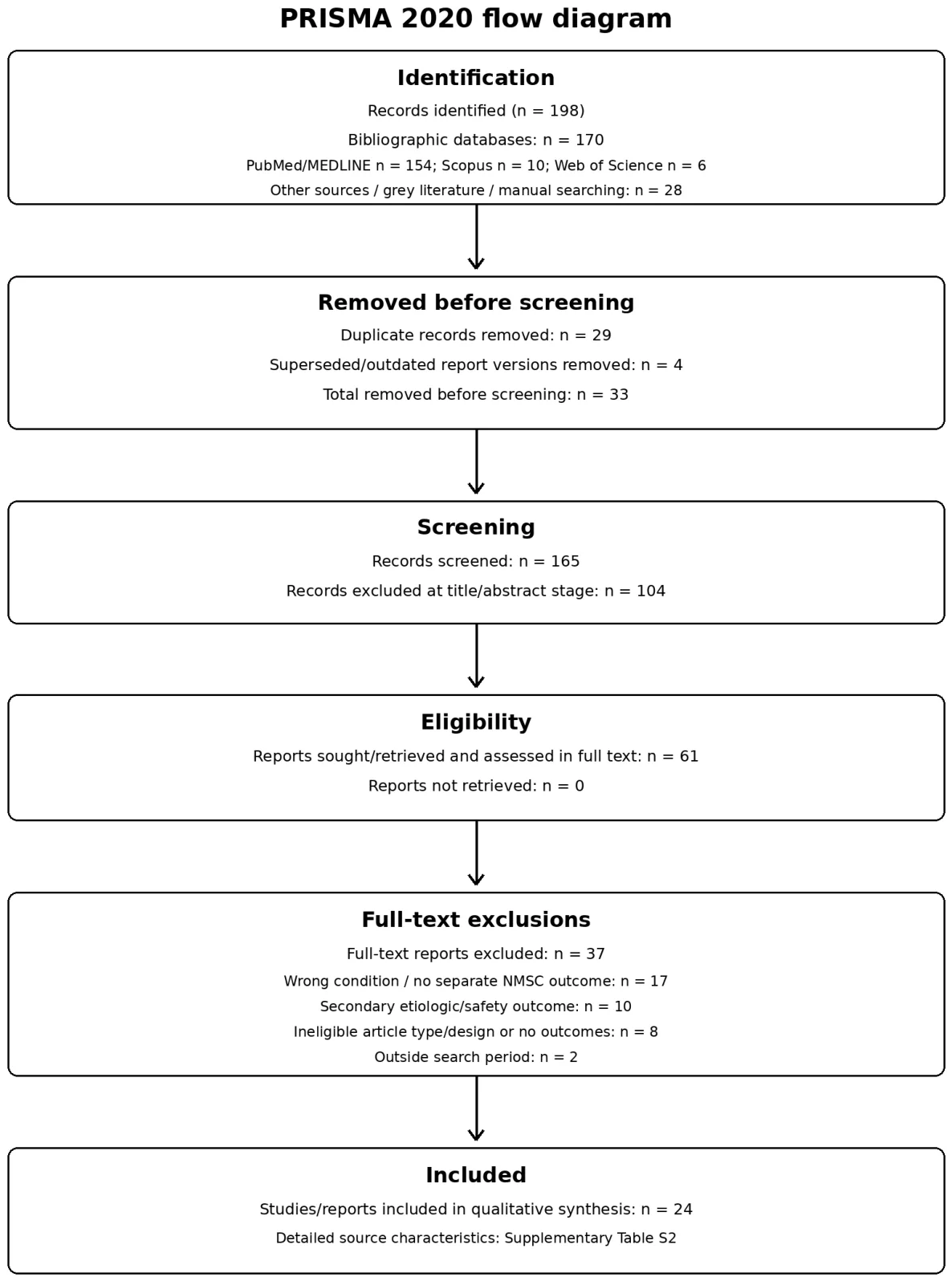
Consolidated PRISMA 2020 selection record following reconciliation of duplicates and full-text eligibility decisions. The figure summarizes the final record set and does not imply that every duplicate was detected before screening.

The consolidated selection record comprised 198 records: 170 from bibliographic databases (PubMed/MEDLINE, 154; Scopus, 10; Web of Science Core Collection, 6) and 28 from registry resources, grey literature, and manual searching. After removal of 29 duplicate records and four superseded report versions, 165 unique records were represented in the screening dataset. Of these, 104 were excluded at title/abstract assessment, and 61 full-text reports were assessed. Thirty-seven reports were excluded, leaving 24 sources in the qualitative synthesis (Figure 1; Supplementary Tables S2 and S3).

The flow diagram presents the reconciled record set rather than the chronological point at which every duplicate was detected. Additional eligibility checks during revision were completed on 30 August 2026. Reports published after the retained 31 March 2026 publication cutoff were not eligible. References retained solely for contextual discussion were not counted among the 24 included sources.

### 3.2. Characteristics of Included Sources

The 24 included sources comprised population-based registry analyses, model-based burden estimates, administrative-data studies, registry reports, and clinical or occupational observational studies. They differed in geographical coverage, observation period, histological detail, tumour counting, rate standardization, and capture of outpatient diagnoses. Studies of selected clinical or occupational groups were interpreted within those populations rather than as national incidence estimates.

Supplementary Table S2 provides characteristics and extracted results for all 24 sources. Supplementary Table S2B presents the descriptive source-level methodological appraisal of exactly the same sources, linked by manuscript reference number. Table 2 is a selected incidence comparison and is not the complete inventory of included evidence. Publications used only for contextual or methodological discussion are not counted among the 24 sources. The included evidence also addressed selected immunosuppressed or occupational populations, screening, pandemic-period care, and site-specific disease [8,16,23,24,25,26,27].

## 4. Epidemiological Synthesis

### 4.1. Registry Architecture as a Determinant of Reported Burden

The included sources showed substantial variation in case ascertainment and tumour counting. Registries differed in whether they distinguished BCC and cSCC, recorded aggregated C44 diagnoses, counted first diagnoses or subsequent primary tumours, and captured outpatient care. Contextual reviews of registration practices support the relevance of these differences [4,6,7]. Multiplicity studies further show that first-person incidence does not capture the full burden of subsequent tumours [15].

Higher recorded rates may reflect more complete ascertainment as well as differences in underlying disease occurrence. Conversely, lower rates may reflect incomplete capture. The relative contribution of these mechanisms cannot be quantified from unharmonized comparisons alone. Source type and ascertainment were therefore considered when interpreting both between-country differences and changes over time; neither a uniformly increasing trend nor a biological geographic gradient was assumed.

### 4.2. Incidence Trends Across Europe

Several population-based registry studies reported increasing BCC or cSCC incidence within defined populations and observation periods [3,10,11,12,13,18]. In Denmark, age-standardized first-diagnosis rates increased from 252 to 338 per 100,000 for BCC and from 49 to 104 per 100,000 for SCC during 2007–2021 [10]. A separate Danish registry study reported increasing cSCC ASIRs during 2005–2023 [11]. Belgian data similarly showed increases in world-standardized BCC rates during 2004–2012 [12]. Spanish multicentre registries reported increasing cSCC ASR-E during 1994–2017 [13]. German findings depended on the observation period: Balkenhol et al. reported a large increase in crude incidence through 2015 and a subsequent plateau, with regional variation [18].

Model-based analyses require separate interpretation. The 28-country GBD analysis described heterogeneous NMSC ASIR patterns, including a slight decline in its overall summary and increases in selected regions and demographic groups [2]. A Spanish GBD-based analysis reported declining ASIRs after 2005 [28]. These patterns cannot be directly equated with trends in individual registries because coverage, endpoints, observation periods, and estimation methods differ. Conversely, increases in selected registries do not establish an increase in all European populations.

Older age groups commonly had a higher incidence, and cSCC generally showed a male predominance in the reported population studies [3,9,10,11,12,13]. An age gradient does not itself demonstrate a change over calendar time. Table 2 presents selected estimates with their source and measurement context; Supplementary Table S2 contains the complete source inventory.

### 4.3. Poland Within the European Context

Polish clinical, administrative, and historical registry sources describe NMSC burden in populations with different coverage and case definitions [1,8,19]. Clinical lesion counts, National Health Fund C44 diagnoses, transplant-recipient data, and historical regional registry counts do not estimate the same outcome. Aggregation under C44 and incomplete capture of outpatient care limit histology-specific and international interpretation; methodological and programme reports provide additional context [4,5,7,20].

These limitations are consistent with under-ascertainment but do not provide a validated correction factor or a precise national estimate of missing cases. Reported counts were therefore treated as recorded burden rather than complete tumour counts or a proven numerical lower bound. Findings from transplant recipients describe a high-risk population and were not extrapolated to the general Polish population [8]. Historical regional data were not used to infer a contemporary nationwide trend [19].

### 4.4. Mortality and the Distinction Between BCC and cSCC

Mortality and survival were less consistently reported than incidence and were retained as distinct outcomes. Registry-based mortality, model-based ASMR, relative survival, and case fatality were not treated as interchangeable measures. The model-based analyses and registry comparisons reported outcomes within their own population and coding definitions [2,3,17,22]. The Spanish cSCC study reported relative survival, which is not an annual population mortality rate [13].

Aggregated NMSC or C44 mortality cannot be interpreted as a histology-specific measure of either BCC or cSCC. Clinical evidence indicates that conventional BCC rarely causes death, whereas cSCC has greater metastatic and mortality relevance [5]. Differences in death certification, coding, competing causes of death, and registry linkage limit cross-country comparisons. The available evidence does not support a single Europe-wide mortality direction inferred from incidence trends.

### 4.5. COVID-19-Related Diagnostic Disruption

Pandemic-period studies documented changes in recorded diagnoses, and the treated case mix in specific settings. Scottish and northern Italian reports described fewer recorded NMSC diagnoses during 2020 [29,30]. These observations concern diagnosis and registration; they do not directly measure a reduction in biological cancer incidence.

A northern Italian surgical study compared two short periods spanning December 2019–January 2020 and December 2020–January 2021 and reported changes in lesion size and histological composition among treated cases [25]. Such selected surgical data cannot establish population incidence or a Europe-wide shift in tumour severity. Changes in access, referral, and prioritization provide plausible explanations, with service reorganization described in a contextual report [31].

The available comparisons do not establish a uniform European post-pandemic rebound or quantify the relative contributions of delayed diagnosis and underlying disease occurrence. Any later increase in recorded diagnoses requires evaluation against local baseline trends, follow-up duration, ascertainment, and the backlog of care.

### 4.6. Determinants of Future Burden

The included occupational and transplant studies describe disease burden in selected exposed or immunosuppressed populations [8,24,26]. They inform the distribution of risk but do not independently quantify future national incidence. The wider literature provides context for cumulative ultraviolet exposure, ageing, and prevention [32,33,34,35].

Future case counts and clinical workload may change with population size and age structure even when age-standardized incidence is stable or declining. Projections are conditional on their demographic, statistical, and ascertainment assumptions and were distinguished from observed registry trends. This review did not calculate a new European forecast.

## 5. Discussion

### 5.1. Principal Findings

The evidence describes a substantial NMSC burden with heterogeneous temporal patterns. Several registries reported increasing BCC or cSCC incidence, while model-based age-standardized estimates varied by geography, demographic group, and period. These findings support source-specific conclusions rather than a single European direction of change.

Data provenance and rate standardization are separate dimensions: a registry-based result may be age-standardized, and a model-based analysis may report absolute counts. Differences between such findings cannot be attributed to modelling or ageing alone. Histological aggregation, tumour-counting rules, outpatient capture, and observation periods also affect interpretation. Polish evidence illustrates these constraints but does not establish the magnitude of national under-registration or an exceptional European trajectory.

### 5.2. Comparison with Previous Evidence

Registry-based increases in defined populations [3,10,11,12,13,18] and heterogeneous model-based patterns [2,28] address partly different questions. The reviewed estimates vary in histology, geography, calendar period, ascertainment, and standardization. A regional crude-rate increase [14] is therefore not directly comparable with a continental model-based ASIR, and a multiplicity study [15] provides evidence about additional tumour burden rather than the direction of national incidence.

The synthesis treats these differences as interpretive constraints. It does not select a preferred direction simply because it is reported by more publications, particularly where datasets overlap. Comparisons with contextual registration reviews [6,7] reinforce the need to identify what each reported measure actually counts.

### 5.3. Interpretation of Between-Country Variation

Between-country variation may reflect both underlying disease occurrence and measurement. Differences in age structure, exposure, population composition, healthcare access, histological verification, and coding can influence recorded rates. Counting all primary tumours produces a different workload measure from counting people with a first diagnosis, even within the same population.

Valid comparisons require compatible case definitions, observation periods, denominators, standard populations, and rules for in situ disease and subsequent primaries. Histology-specific person-based incidence and tumour-based counts provide complementary information. The available heterogeneous estimates do not support simple country rankings or quantification of the biological component of geographic differences.

### 5.4. Implications for Surveillance and Prevention

Surveillance priorities are separate reporting of BCC and cSCC, an explicit distinction between first and subsequent primary tumours, and improved linkage of pathology with outpatient and other care data. Person-based incidence and tumour-based workload are complementary indicators. Registration reports also need to identify standard populations, case definitions, and changes in coverage that may affect temporal comparisons.

The distribution of disease supports attention to older and immunosuppressed populations, including transplant recipients [8,26], and to occupational ultraviolet exposure [24]. Clinical and prevention references inform risk-adapted follow-up and ultraviolet protection [5,32,33,34,35]. This epidemiological synthesis does not itself establish the effectiveness or optimal interval of screening or follow-up programmes.

Service planning requires absolute case numbers, repeated procedures, multiplicity, and morbidity as well as standardized rates. Stable or declining ASIRs can coexist with increasing workload as populations age or grow. Forecasts therefore require explicit demographic and registration assumptions; the review does not provide a single European cost estimate or a newly calculated burden projection.

### 5.5. Strengths and Limitations

The review brings together epidemiological results and information about how they were generated. Supplementary Tables S2 and S2B make the included sources and their methodological concern judgments visible, while the synthesis distinguishes source type, outcome definition, and rate standardization.

Several limitations affect interpretation. Designs, population coverage, histology, tumour-counting rules, and standard populations differed substantially. English- and Polish-language restrictions and geographically uneven supplementary searches may have introduced language and availability bias. Some sources shared underlying registry or model inputs, so agreement did not necessarily represent independent corroboration. Clinical and occupational samples were not representative of all national populations. Model-based estimates inherited limitations of their input data and assumptions; registry-based estimates were also susceptible to under-registration and changes in ascertainment.

The source-level methodological appraisal was a structured descriptive framework, not a validated risk-of-bias tool or formal certainty grading system. Its overall judgments depend on the purpose for which a source is interpreted. The review was not prospectively registered, and no publicly accessible protocol was prepared. Short pandemic-period comparisons could not distinguish all effects of delayed diagnosis, care selection, and underlying incidence. These limitations constrain both the magnitude and direction of cross-source temporal comparisons and do not justify a uniform Europe-wide incidence trend. The targeted revision checks did not constitute a new exhaustive database search with an extended publication window.

## 6. Conclusions

NMSC represents a substantial and incompletely captured cancer burden in Europe. Registry-based studies document increases in BCC or cSCC incidence in several defined populations, while model-based age-standardized trends differ by geography, sex, age, and observation period. Neither finding establishes a uniform European trend, and changes in case counts are not interchangeable with changes in standardized rates.

Polish evidence is constrained by heterogeneous ascertainment and aggregation of histological categories, limiting estimates of total burden and international comparison. Histology-specific registration, transparent counting of first and subsequent primaries, and improved outpatient capture are priorities for surveillance and service planning. Pandemic-period reductions in recorded diagnoses require interpretation as changes in observed diagnostic activity, without assuming a corresponding biological decline or a demonstrated Europe-wide rebound.

## Figures and Tables

**Table 2:** Selected European incidence findings by source type and measure. Rates are not pooled because population coverage, case definitions, standard populations, observation periods, and counting rules differ. Supplementary Table S2 contains the complete inventory of included sources. A trend estimate not reproduced in this selected table is not necessarily absent from the original article. Arrows (→) indicate change from the earlier to the later reported value and are not reference citations.

Population and Period	Source and Endpoint	Rate or Direction	Trend Measure	Interpretation
Europe, 28 countries; 1992–2021 [2]	GBD model; NMSC	Heterogeneous ASIR patterns	Overall summary slightly declining; regional and demographic increases	Model-based estimates; not observed tumour-level registry counts.
Denmark; 2007–2021 [10]	Registry; first BCC	252 → 338 per 100,000	Endpoint comparison; no AAPC reproduced	Age-standardized to ESP 2013; first diagnoses.
Denmark; 2007–2021 [10]	Registry; first SCC	49 → 104 per 100,000	Endpoint comparison; no AAPC reproduced	Age-standardized to ESP 2013; first diagnoses.
Denmark; 2005–2023 [11]	Registry; first cSCC	2023 ASIR: male 131.6; female 77.7 per 100,000 person years	EAPC: male +2.6%; female +3.1%	Individuals aged ≥20 years; invasive cSCC separated from CIS and keratoacanthoma.
Four Spanish registries; 1994–2017 [13]	Registry; first invasive cSCC	Period ASR-E: male 56.6; female 25.0 per 100,000	Both sexes APC +2.0% (95% CI 1.6–2.4)	ESP 2013. Combined APC is not a separate estimate for each sex.
Germany; 1986–2019 [18]	Registry; first cSCC	Crude incidence increased >500% during 1986–2015	Later plateau, with regional variation	The >500% change concerns crude rates, not ASIR; no national or European extrapolation.
German regions, Netherlands, Scotland; 1989/90–2020 [3]	Registry; cSCC	ASIR increased in the populations studied	Source-reported annual increases 2.4–5.7%	ESP 2013; source-specific periods; historical findings separated from projections.
Belgium; 2004–2012 [12]	Registry; BCC	Male: 36.9 → 98.4; female: 34.2 → 102.0 per 100,000 person years	Endpoint comparison; no annual estimate reproduced	World-standardized rates; multiple primary tumours included; registration evolved.
Spain; 1990–2019 [28]	GBD/GHDx model; NMSC	Declining ASIR after 2005	No numerical trend estimate reproduced	Model-based national estimates; not interchangeable with rates from selected Spanish registries.

## Data Availability

No new primary data were generated. The search strategies, extraction matrix, source-level methodological appraisal, and full-text exclusion list are provided in Supplementary Methods S1 and Supplementary Tables S2, S2B and S3.

## Supplementary Materials

The following supporting information can be downloaded at: https://www.mdpi.com/article/10.3390/cancers18182970/s1, Supplementary Methods S1: Reported Database-Specific Search Strategies; Supplementary Table S1: PRISMA 2020 Checklist [21]; Supplementary Table S2: Characteristics and Extracted Results of the 24 Included Sources [1,2,3,8,9,10,11,12,13,14,15,16,17,18,19,22,23,24,25,26,27,28,29,30]; Supplementary Table S2B: Descriptive Source-Level Methodological Appraisal of the 24 Included Sources, including domain-level and overall methodological concern judgments and rationales [1,2,3,8,9,10,11,12,13,14,15,16,17,18,19,22,23,24,25,26,27,28,29,30]; Supplementary Table S3: Full-Text Reports Excluded after Eligibility Assessment, with one principal reason per report.

## Abbreviations

AAPC	average annual percentage change
APC/EAPC	annual/estimated annual percentage change
ASMR	age-standardized mortality rate
ASIR	age-standardized incidence rate
ASR	age-standardized rate
ASR-E	European age-standardized rate
BCC	basal cell carcinoma
CHEK2	gene producing the checkpoint kinase 2 protein.
cSCC	cutaneous squamous cell carcinoma
EAPC	estimated annual percentage change
ECIS	European Cancer Information System
GLOBOCAN	Global Cancer Observatory
IARC	International Agency for Research on Cancer
ICD-10	International Classification of Diseases
NCRR	National Cancer Registry Reports (Polish National Cancer Registry)
NMSC	non-melanoma skin cancer
UVR	ultraviolet radiation
