# Supplementary material for: Epidemiology of Non-Melanoma Skin Cancer in Europe over the Last Decade: A Systematic Review
Source: Cancers (Basel). 2026 Sep 14;18(18):2970. doi: 10.3390/cancers18182970 (PMC13605927; doi:10.3390/cancers18182970)
Supplement: Supplementary file 1 [file cancers-18-02970-s001.zip › Supplementary_Methods_S1.pdf]

Supplementary Methods S1. Reported Database-Specific Search Strategies

Manuscript: Epidemiology of Non-Melanoma Skin Cancer in Europe over the Last Decade: A Systematic Review

Initial search date: 31 March 2026. Last supplementary eligibility check during revision: 30 August 2026.

Eligibility limits applied to the bibliographic search: publication period 1 January 2015 to 31 March 2026; languages English or Polish. This window concerns publication dates, not the observation periods of the underlying epidemiological data. Historical observation periods were eligible when the publication met the date criterion.

Databases: PubMed/MEDLINE, Scopus, and Web of Science Core Collection.

Search approach: Controlled vocabulary was used where available and combined with free-text terms for non-melanoma skin cancer/keratinocyte carcinoma, epidemiological outcomes, and European geography. No automation tools were used for study selection.

1. PubMed/MEDLINE

Search date: 31 March 2026

Filters/limits: Publication date 1 January 2015–31 March 2026; English or Polish.

Records retrieved: n = 154

Reported search string:

```
("Skin Neoplasms"[Mesh] OR "Carcinoma, Basal Cell"[Mesh] OR "Carcinoma, Squamous Cell"[Mesh] OR "non-
melanoma skin cancer"[Title/Abstract] OR "nonmelanoma skin cancer"[Title/Abstract] OR "keratinocyte
carcinoma"[Title/Abstract] OR "basal cell carcinoma"[Title/Abstract] OR BCC[Title/Abstract] OR
"cutaneous squamous cell carcinoma"[Title/Abstract] OR cSCC[Title/Abstract]) AND
(epidemiology[Title/Abstract] OR incidence[Title/Abstract] OR prevalence[Title/Abstract] OR
mortality[Title/Abstract] OR trend*[Title/Abstract] OR registry[Title/Abstract] OR
registries[Title/Abstract] OR "disease burden"[Title/Abstract]) AND (Europe[Title/Abstract] OR
European[Title/Abstract] OR "Albania"[Title/Abstract] OR "Andorra"[Title/Abstract] OR
"Armenia"[Title/Abstract] OR "Austria"[Title/Abstract] OR "Azerbaijan"[Title/Abstract] OR
"Belarus"[Title/Abstract] OR "Belgium"[Title/Abstract] OR "Bosnia and Herzegovina"[Title/Abstract] OR
"Bulgaria"[Title/Abstract] OR "Croatia"[Title/Abstract] OR "Cyprus"[Title/Abstract] OR
"Czechia"[Title/Abstract] OR "Czech Republic"[Title/Abstract] OR "Denmark"[Title/Abstract] OR
"Estonia"[Title/Abstract] OR "Finland"[Title/Abstract] OR "France"[Title/Abstract] OR
"Georgia"[Title/Abstract] OR "Germany"[Title/Abstract] OR "Greece"[Title/Abstract] OR
"Hungary"[Title/Abstract] OR "Iceland"[Title/Abstract] OR "Ireland"[Title/Abstract] OR
"Italy"[Title/Abstract] OR "Kosovo"[Title/Abstract] OR "Latvia"[Title/Abstract] OR
"Liechtenstein"[Title/Abstract] OR "Lithuania"[Title/Abstract] OR "Luxembourg"[Title/Abstract] OR
"Malta"[Title/Abstract] OR "Moldova"[Title/Abstract] OR "Republic of Moldova"[Title/Abstract] OR
"Monaco"[Title/Abstract] OR "Montenegro"[Title/Abstract] OR "Netherlands"[Title/Abstract] OR "North
Macedonia"[Title/Abstract] OR "Macedonia"[Title/Abstract] OR "Norway"[Title/Abstract] OR
"Poland"[Title/Abstract] OR "Portugal"[Title/Abstract] OR "Romania"[Title/Abstract] OR
"Russia"[Title/Abstract] OR "Russian Federation"[Title/Abstract] OR "San Marino"[Title/Abstract] OR
"Serbia"[Title/Abstract] OR "Slovakia"[Title/Abstract] OR "Slovenia"[Title/Abstract] OR
"Spain"[Title/Abstract] OR "Sweden"[Title/Abstract] OR "Switzerland"[Title/Abstract] OR
"Turkey"[Title/Abstract] OR "Türkiye"[Title/Abstract] OR "Ukraine"[Title/Abstract] OR "United
Kingdom"[Title/Abstract] OR "UK"[Title/Abstract] OR "Great Britain"[Title/Abstract] OR
"England"[Title/Abstract] OR "Scotland"[Title/Abstract] OR "Wales"[Title/Abstract] OR "Northern
Ireland"[Title/Abstract] OR "Vatican City"[Title/Abstract]) AND ("2015/01/01"[Date - Publication] :
"2026/03/31"[Date - Publication])
```

2. Scopus

Search date: 31 March 2026

Filters/limits: PUBYEAR > 2014 AND PUBYEAR < 2027; language restricted to English or Polish. The exact publication-date criterion was applied during eligibility assessment.

Records retrieved: n = 10

Reported search string:

```
TITLE-ABS-KEY("non-melanoma skin cancer" OR "nonmelanoma skin cancer" OR "keratinocyte carcinoma" OR "basal cell carcinoma" OR BCC OR "cutaneous squamous cell carcinoma" OR cSCC) AND TITLE-ABS-KEY(epidemiology OR incidence OR prevalence OR mortality OR trend* OR registr* OR "disease burden") AND TITLE-ABS-KEY(Europe OR European OR "Albania" OR "Andorra" OR "Armenia" OR "Austria" OR "Azerbaijan" OR "Belarus" OR "Belgium" OR "Bosnia and Herzegovina" OR "Bulgaria" OR "Croatia" OR "Cyprus" OR "Czechia" OR "Czech Republic" OR "Denmark" OR "Estonia" OR "Finland" OR "France" OR "Georgia" OR "Germany" OR "Greece" OR "Hungary" OR "Iceland" OR "Ireland" OR "Italy" OR "Kosovo" OR "Latvia" OR "Liechtenstein" OR "Lithuania" OR "Luxembourg" OR "Malta" OR "Moldova" OR "Republic of Moldova" OR "Monaco" OR "Montenegro" OR "Netherlands" OR "North Macedonia" OR "Macedonia" OR "Norway" OR "Poland" OR "Portugal" OR "Romania" OR "Russia" OR "Russian Federation" OR "San Marino" OR "Serbia" OR "Slovakia" OR "Slovenia" OR "Spain" OR "Sweden" OR "Switzerland" OR "Turkey" OR "Türkiye" OR "Ukraine" OR "United Kingdom" OR "UK" OR "Great Britain" OR "England" OR "Scotland" OR "Wales" OR "Northern Ireland" OR "Vatican City") AND PUBYEAR > 2014 AND PUBYEAR < 2027
```

Database-specific syntax: TITLE-ABS-KEY was used for title/abstract/keyword searching; PUBYEAR limits were applied in Scopus syntax.

3. Web of Science Core Collection

Search date: 31 March 2026

Filters/limits: Timespan 2015–2026; languages English or Polish. The exact publication-date criterion was applied during eligibility assessment.

Records retrieved: n = 6

Reported search string:

```
TS=("non-melanoma skin cancer" OR "nonmelanoma skin cancer" OR "keratinocyte carcinoma" OR "basal cell carcinoma" OR BCC OR "cutaneous squamous cell carcinoma" OR cSCC) AND TS=(epidemiology OR incidence OR prevalence OR mortality OR trend* OR registr* OR "disease burden") AND TS=(Europe OR European OR "Albania" OR "Andorra" OR "Armenia" OR "Austria" OR "Azerbaijan" OR "Belarus" OR "Belgium" OR "Bosnia and Herzegovina" OR "Bulgaria" OR "Croatia" OR "Cyprus" OR "Czechia" OR "Czech Republic" OR "Denmark" OR "Estonia" OR "Finland" OR "France" OR "Georgia" OR "Germany" OR "Greece" OR "Hungary" OR "Iceland" OR "Ireland" OR "Italy" OR "Kosovo" OR "Latvia" OR "Liechtenstein" OR "Lithuania" OR "Luxembourg" OR "Malta" OR "Moldova" OR "Republic of Moldova" OR "Monaco" OR "Montenegro" OR "Netherlands" OR "North Macedonia" OR "Macedonia" OR "Norway" OR "Poland" OR "Portugal" OR "Romania" OR "Russia" OR "Russian Federation" OR "San Marino" OR "Serbia" OR "Slovakia" OR "Slovenia" OR "Spain" OR "Sweden" OR "Switzerland" OR "Turkey" OR "Türkiye" OR "Ukraine" OR "United Kingdom" OR "UK" OR "Great Britain" OR "England" OR "Scotland" OR "Wales" OR "Northern Ireland" OR "Vatican City")
```

Database-specific syntax: TS= was used for Topic searching (title, abstract, author keywords, and Keywords Plus, according to platform indexing).

4. Supplementary Sources and Grey Literature

Sources searched or verified: Polish National Cancer Registry and the 'Malignant Tumours in Poland' reports; European Cancer Information System (ECIS); Global Cancer Observatory (GLOBOCAN/IARC); reference lists of eligible publications and relevant reviews.

Search date: 31 March 2026

Search terms: combinations of “non-melanoma skin cancer”, “nonmelanoma skin cancer”, “keratinocyte carcinoma”, “skin cancer”, “C44”, “basal cell carcinoma”, “cutaneous squamous cell carcinoma”, “incidence”, “prevalence”, “mortality”, “trend”, “registry”, “Poland”, and “Europe”.

Procedure: Supplementary-source searching was targeted rather than a standardized exhaustive search of every national European cancer-registry website. Reference lists of eligible papers and relevant reviews were screened manually. This asymmetry in grey-literature retrieval is acknowledged in the manuscript as a potential source of geographical availability bias.

5. Search Yield

Source	Records retrieved
PubMed/MEDLINE	154
Scopus	10
Web of Science Core Collection	6
Total bibliographic database records	170
Registry reports / grey literature / manual searching	28
Total records identified	198

6. European Geographic Terms Used in the Search

To make the geographic component reproducible, the search syntax explicitly includes the following European country names and commonly used English name variants:

Albania	Andorra	Armenia
Austria	Azerbaijan	Belarus
Belgium	Bosnia and Herzegovina	Bulgaria
Croatia	Cyprus	Czechia
Czech Republic	Denmark	Estonia
Finland	France	Georgia
Germany	Greece	Hungary
Iceland	Ireland	Italy
Kosovo	Latvia	Liechtenstein
Lithuania	Luxembourg	Malta
Moldova	Republic of Moldova	Monaco
Montenegro	Netherlands	North Macedonia
Macedonia	Norway	Poland
Portugal	Romania	Russia
Russian Federation	San Marino	Serbia
Slovakia	Slovenia	Spain
Sweden	Switzerland	Turkey
Türkiye	Ukraine	United Kingdom
UK	Great Britain	England
Scotland	Wales	Northern Ireland
Vatican City		

7. Reproducibility and Deviations

No automation tools were used for title/abstract screening, full-text eligibility assessment, or data extraction.

The strategies use platform-specific field tags and date syntax; PubMed additionally uses controlled vocabulary. Searchable fields and term coverage are not identical across databases.

Registration/protocol: The review was not prospectively registered, and no publicly accessible protocol was prepared. Additional literature checks during manuscript revision continued until 30 August 2026 and assessed records for inclusion under the retained publication-date criterion of 1 January 2015–31 March 2026. These checks were targeted and are not presented as a complete rerun of all three databases or an extension of the publication eligibility window.

The yield table presents the consolidated counts reported in Figure 1, not a separate count of the initial search and revision checks. The supplied documentation does not allocate additional records to individual revision dates or show which source totals changed after 31 March 2026.
