# Supplementary material for: Epidemiology of Non-Melanoma Skin Cancer in Europe over the Last Decade: A Systematic Review
Source: Cancers (Basel). 2026 Sep 14;18(18):2970. doi: 10.3390/cancers18182970 (PMC13605927; doi:10.3390/cancers18182970)
Supplement: Supplementary file 1 [file cancers-18-02970-s001.zip › Supplementary_Table_S1.pdf]

Supplementary Table S1. PRISMA 2020 Checklist

Manuscript: Epidemiology of Non-Melanoma Skin Cancer in Europe over the Last Decade: A Systematic Review

Consolidated selection record: 198 identified; 33 removed; 165 screened; 104 excluded at title/abstract stage; 61 full-text assessments; 37 full-text exclusions; 24 included sources.

Item	PRISMA 2020 topic	Location in submitted materials	Reporting status / outstanding information
1	Title	Title page	Identifies the report as a systematic review.
2	Abstract	Abstract	Structured abstract supplied; no prospective registration or funding statement is included in the abstract. Do not equate this map with full PRISMA abstract compliance.
3	Rationale	Introduction	Rationale emphasizes the European NMSC burden and the effect of heterogeneous registration practices on reported incidence.
4	Objectives	Introduction / review objective	Objectives specify incidence, mortality, demographic distribution, registry methodology and COVID-19-related diagnostic disruption.
5	Eligibility criteria	Methods 2.2	Population/setting, condition, outcomes, study/report types, publication period, language and exclusion criteria are specified.
6	Information sources	Methods 2.3; Supplementary Methods S1	Initial and targeted-check dates are distinguished. Exact revision-check sources/dates/yields and full grey-literature search logs require author confirmation in S1.
7	Search strategy	Methods 2.4; Supplementary Methods S1	Reported database-specific strings are present. Historical execution, language-filter application and WoS indexes remain to be verified; see S1 section 8.
8	Selection process	Methods 2.5	Collaborative screening by five authors is stated. Number of assessors per record, independence and disagreement resolution are not specified and require confirmation.
9	Data collection process	Methods 2.6	Collaborative extraction is stated. Number of extractors per report, independence and disagreement handling require confirmation.
10a	Data items: outcomes	Methods 2.6	Extracted items include country/region, study period, subtype/case definition, source, multiple-primary rules, case counts, crude/standardized incidence, mortality, APC/EAPC/AAPC, age/sex and completeness indicators.
10b	Data items: other variables	Methods 2.6	COVID-19 diagnostic disruption, registry design and ascertainment variables were extracted; missing values were not imputed.
11	Study risk of bias	Methods 2.7; Supplementary Table S2B	Descriptive source-level methodological appraisal, not a validated RoB tool. Operational domain criteria, evidence locators and assessor procedure are incomplete.
12	Effect measures	Methods 2.8	Case counts, crude rates, ASIR/ASMR and author-reported APC/EAPC/AAPC were used; author-derived two-point annual changes were removed.
13a	Synthesis eligibility	Methods 2.8	Sources meeting the stated eligibility criteria were grouped by source type, endpoint, population and period.
13b	Data preparation	Methods 2.6 and 2.8	No missing epidemiological values were imputed; source-reported measures were retained in their original methodological context.
13c	Tabulation / display	Table 2; Supplementary Tables S2 and S2B	Comparative results are tabulated without pooling; source characteristics and methodological concern are provided at source level.

Item	PRISMA 2020 topic	Location in submitted materials	Reporting status / outstanding information
13d	Synthesis methods	Methods 2.8	Narrative/descriptive comparative synthesis was used; meta-analysis was not undertaken because of major heterogeneity in definitions, coverage, standard populations and tumour-counting rules.
13e	Heterogeneity exploration	Section 4.1–4.6; Discussion	Heterogeneity is interpreted by registry architecture, tumour subtype, geography, age/sex, multiple-primary rules and pandemic-related service disruption.
13f	Sensitivity analyses	Methods 2.8	No formal sensitivity analyses were undertaken, as explicitly stated in Methods 2.8.
14	Reporting bias assessment	Methods 2.9; Discussion 5.5	Reporting bias was considered qualitatively. No statistical small-study-effects assessment was performed; heterogeneous outcomes precluded a meaningful common analysis.
15	Certainty assessment	Methods 2.9; Discussion	No formal certainty grading; methodological limitations and comparability informed interpretation.
16a	Study selection	Results 3.1; Figure 1; S1; S3	Counts reconcile arithmetically. Search/screening logs, zero unretrieved reports and row-to-category allocation of exclusions remain unverified.
16b	Excluded studies	Supplementary Table S3	Exactly 37 unique report rows are listed. Principal reasons are preserved; author confirmation is required for PRISMA category allocation and consistent eligibility decisions.
17	Study characteristics	Results 3.2; Supplementary Table S2	All 24 source identities are present. Some observation dates, quantitative endpoints and source locators remain incomplete; see S2 author-confirmation notes.
18	Risk of bias in studies	Supplementary Table S2B	All 24 sources have nine domain judgments, overall concern and a rationale. Ratings are descriptive and require source-level verification; no validated RoB assessment is claimed.
19	Results of individual studies	Section 4; Table 2; Supplementary Table S2	Individual findings are supplied, but several rows lack numerical NMSC endpoints and systematic source locators. Full extraction completeness is not established.
20a	Results of syntheses	Section 4.1–4.6	Main patterns are synthesized narratively; registry-based increases are distinguished from heterogeneous model-based age-standardized trends.
20b	Statistical synthesis	Not applicable	No meta-analysis or pooled effect estimate was calculated.
20c	Investigations of heterogeneity	Section 4.1–4.6; Discussion 5.3	Differences in registration, histology, multiple-primary rules, age, sex, geography and pandemic disruption are explicitly evaluated.
20d	Sensitivity analyses	Methods 2.8	No formal sensitivity analyses were undertaken, as explicitly stated in Methods 2.8.
21	Reporting biases	Discussion 5.5	Language restriction, uneven grey-literature search intensity, publication/availability bias and incomplete registry capture are acknowledged.
22	Certainty of evidence	Methods 2.9; Discussion 5.1 and 5.5	Conclusions are qualified according to consistency and data quality; uniform Europe-wide incidence increase is not claimed.
23a	Interpretation	Discussion 5.1–5.4	Findings are interpreted in relation to previous evidence and registry methodology.
23b	Limitations of evidence	Discussion 5.5	Heterogeneity, incomplete registry capture, overlap, institutional-report quality and non-comparability of standardized rates are discussed.
23c	Limitations of review processes	Discussion 5.5	English/Polish restriction and geographically uneven supplementary-source searching are acknowledged.

Item	PRISMA 2020 topic	Location in submitted materials	Reporting status / outstanding information
23d	Implications	Discussion 5.4; Conclusions	Implications emphasize histology-specific registration, tumour-aware surveillance, UV prevention, high-risk follow-up and improved outpatient/pathology linkage.
24a	Registration	Methods 2.1	The review was not prospectively registered.
24b	Protocol	Methods 2.1	No publicly accessible protocol was prepared.
24c	Amendments	Methods 2.1, 2.3–2.4; Results 3.1; S1	No prospective registration or publicly accessible protocol is reported. Targeted revision checks are disclosed; confirm the actual retained cutoff and any eligibility amendments.
25	Support	Funding statement; Acknowledgments	No external funding is declared; institutional academic support is acknowledged.
26	Competing interests	Conflicts of Interest	The authors declare no conflict of interest.
27	Availability of data, code and materials	Data Availability Statement; Supplementary Materials	The supplied package contains S1 and S2/S2B/S3. Public or editor/reviewer accessibility after upload has not been tested; original search exports, source files and complete appraisal evidence are not supplied.

Adapted reporting map for PRISMA 2020, including all 42 items/subitems. Source: Page MJ et al. BMJ 2021;372:n71. doi:10.1136/bmj.n71. Official checklist: https://www.prisma-statement.org/prisma-2020-checklist (CC BY 4.0). This map identifies reporting gaps and does not certify complete compliance.
