# Supplementary material for: Epidemiology of Non-Melanoma Skin Cancer in Europe over the Last Decade: A Systematic Review
Source: Cancers (Basel). 2026 Sep 14;18(18):2970. doi: 10.3390/cancers18182970 (PMC13605927; doi:10.3390/cancers18182970)
Supplement: Supplementary file 1 [file cancers-18-02970-s001.zip › Supplementary_Table_S2.pdf]

Supplementary Table S2. Characteristics and Extracted Results of the 24 Included Sources

Characteristics and extracted results of the 24 included sources. Cells identify outcomes not evaluated or outside the study design; missing values were not imputed.

Reference	Country/region & period	Design; population; data source	Case definition / ascertainment	Multiple tumours / in situ	Key quantitative epidemiological results	Mortality / survival	Main methodological limitations
1. Ciążyńska et al., Scientific Reports 2021;11:4337. doi:10.1038/s41598-021-83502-8	Poland (7 centres; national NHF data used for temporal context) Study period: 1999-2019	Retrospective multicentre clinicopathological study with analysis of National Health Fund C44 data Population: 10,083 patients; 13,913 histologically confirmed NMSC lesions (11,848 BCC; 2,065 cSCC) Data source: Seven clinical centres plus Polish National Health Fund administrative data	BCC and cSCC; excised lesions histologically confirmed. NHF trend based on ICD-10 C44 diagnoses.	Tumour-based clinical series included multiple lesions; 23% of patients had >1 NMSC. In situ disease was not the target outcome.	Increasing C44 diagnosis rate; annual growth approximately 5-7% in 2005-2014 and <2% annually after 2014. BCC:cSCC ratio 5.7:1.	Mortality not evaluated.	Clinical centres were not a complete population registry; national C44 data aggregate histologies and may incompletely capture outpatient lesions.
2. Sendín-Martín et al., Clin Transl Oncol. 2026;28:302-319. doi:10.1007/s12094-025-03985-z	28 European countries Study period: 1992-2021	Longitudinal ecological analysis Population: European populations represented in Global Burden of Disease estimates; >27 million estimated NMSC cases Data source: Global Burden of Disease database	NMSC, BCC and SCC estimates; incidence and SCC mortality analysed by sex, age and European region.	Model-based disease estimates rather than tumour-level registry counting; in situ tumours not separately analysed.	Estimated NMSC burden over 1992–2021: 27,143,489 cases (86% BCC, 14% SCC). Model-based ASIR patterns were heterogeneous: an overall slight decline was described alongside modest increases in Central/Eastern Europe. These are not tumour-level observed registry counts.	570,525 estimated SCC deaths; overall ASMR declined in women, but increased in selected older male groups in Northern/Western Europe.	GBD modelling depends on input data quality and assumptions; between-country differences partly reflect registry completeness and coding.
3. Keim et al., Eur J Cancer. 2023;183:60-68. doi:10.1016/j.ejca.2023.01.017	Germany (Saarland, Schleswig-Holstein), Netherlands, Scotland Study period: Approximately 1989/1990-2020	Comparative population-based registry trend study Population: Residents covered by four cancer registries Data source: Population cancer registries	Registry-recorded invasive cSCC; histology-specific incidence analysed separately from aggregate C44.	Registry-specific rules differed; study explicitly compared registration practices. In situ disease was not the principal cSCC incidence endpoint.	Age-standardised cSCC incidence increased in all registries; annual increases approximately 2.4-5.7%; projections indicated further growth to 2044.	cSCC mortality trends were analysed and were substantially lower than incidence; sex- and registry-specific patterns differed.	Cross-registry heterogeneity in completeness, multiple-primary rules and historical coding limits absolute comparisons.
8. Wysocki et al., Adv Clin Exp Med. 2025;34:901-909. doi:10.17219/acem/199653	Poland Study period: 2010-2022	Nationwide real-world retrospective cohort study Population: 17,207 solid-organ transplant recipients (kidney, liver or heart) Data source: Polish National Health Fund administrative database; comparison with general Polish population	NMSC with BCC and cSCC analysed in immunosuppressed transplant recipients using diagnostic/claims data.	Patient-based administrative ascertainment; histopathological verification and multiple-primary counting were not uniformly available in claims data; in situ tumours not a principal endpoint.	Markedly elevated NMSC burden in transplant recipients; cSCC risk particularly increased under chronic immunosuppression.	NMSC-specific mortality not the main outcome.	Claims-based case definition, potential coding misclassification, and limited tumour-level pathology/staging information.
9. Bartoš & Kullová, Klin Onkol. 2018;31:40-45. doi:10.14735/amko201840	Žilina, Slovakia Study period: Retrospective clinical series; publication 2018	Retrospective clinicopathological comparative study Population: 541 patients with 719 BCCs and 126 patients with 162 cSCCs Data source: Department of Pathology and Dermatovenerology, Faculty Hospital Žilina	Histologically diagnosed BCC and cSCC.	Tumour-based counts allowed multiple lesions per patient; invasive BCC/cSCC analysed; in situ lesions were not the study focus.	cSCC occurred more often in men (68.2%); mean age 70.8 years for BCC and 78.2 years for cSCC; head/neck location 69.8% and 81.4%, respectively.	Mortality not evaluated.	Single-centre/clinical referral series; no population denominator, therefore no population incidence rate.
10. Krogh Holdam et al., Cancer Epidemiol. 2025;94:102732. doi:10.1016/j.canep.2024.102732	Denmark Study period: 2007-2021	Nationwide population-based register study Population: 183,338 patients with first BCC and 42,233 with first SCC Data source: Population-based Danish databases	Incident BCC and SCC; age-adjusted incidence and tumour characteristics.	First-time incidence was the principal person-based endpoint; larger/invasive tumours and head/neck tumours separately characterised; in situ disease not the principal endpoint.	Age-adjusted incidence rose from 252 to 338/100,000 for BCC and 49 to 104/100,000 for SCC (ESP 2013).	Mortality not evaluated.	First-incidence approach underestimates total tumour workload from multiple primary keratinocyte carcinomas.

Reference	Country/region & period	Design; population; data source	Case definition / ascertainment	Multiple tumours / in situ	Key quantitative epidemiological results	Mortality / survival	Main methodological limitations
11. Duffau et al., JAMA Dermatol. 2026;162:333-342. doi:10.1001/jamadermatol.2025.5700	Denmark<br>Study period: 2005-2023	Nationwide population-based registry cohort<br>Population: 95,352 individuals; 109,787 first-time histologically confirmed keratinocyte neoplasms<br>Data source: Danish Pathology Registry and Danish Cancer Registry	First-time cSCC, cSCC in situ (CIS) and keratoacanthoma, all histologically confirmed.	First-time diagnosis for each defined entity; invasive cSCC and CIS explicitly separated.	In 2023, cSCC ASIRs reached 131.6 and 77.7 per 100,000 person-years in male and female individuals, respectively; EAPCs during 2005–2023 were +2.6% and +3.1%. CIS increased more rapidly. A published correction addressed figure labels and keys.	Mortality not evaluated.	Focus on first-time diagnoses does not quantify all subsequent primary cSCCs; registry definitions may not match other countries.
12. Callens et al., J Eur Acad Dermatol Venereol. 2016;30:1912-1918. doi:10.1111/jdv.13703	Belgium<br>Study period: 2004-2012	Population-based registry study<br>Population: 130,339 patients; 146,407 tumours (113,254 BCC; 33,153 SCC)<br>Data source: Belgian Cancer Registry, predominantly pathology notifications; compulsory registration	BCC and SCC identified through cancer/pathology registration.	Multiple primary skin tumours were included; ~10% of patients had multiple tumours. In situ SCC was not the main invasive SCC endpoint.	World-standardised BCC rates rose from 36.9 to 98.4/100,000 in men and 34.2 to 102.0 in women; SCC from 14.9 to 24.7 in men and 6.8 to 13.5 in women.	Mortality not evaluated.	Registration completeness changed over time; pathology-driven ascertainment may differ from registry systems in other countries.
13. Fernández-Martínez et al., Actas Dermosifiliogr. 2026;117:104490. doi:10.1016/j.ad.2025.104490	Spain (Girona, Granada, Navarre, Tarragona)<br>Study period: 1994-2017	Retrospective multicentre population-based cancer-registry study<br>Population: 22,434 first incident cSCC cases; 61.5% men<br>Data source: Four Spanish population-based cancer registries	First incident invasive cSCC registered in participating registries.	First cSCC per person for incidence analysis; in situ disease excluded from the invasive cSCC endpoint.	Period ASR-E (1994–2017): 56.6/100,000 in men and 25.0/100,000 in women (ESP 2013). Overall APC for both sexes combined: +2.0% (95% CI 1.6–2.4). The overall APC is not a sex-specific estimate.	Five-year relative survival 93.5%; mortality rate itself was not the main reported measure.	Only Spanish registries that register cSCC were included; therefore national coverage was incomplete.
14. Santos-Juanes et al., J Clin Med. 2025;14:5654. doi:10.3390/jcm14165654	Asturias, Spain<br>Study period: 2014-2023	Retrospective pathology-registry incidence study<br>Population: Catchment population ~350,000/year; 7,477 BCC and 2,689 SCC (plus melanoma cases)<br>Data source: Central University Hospital of Asturias pathology registry plus municipal population denominators	Pathologically diagnosed BCC and SCC; tumour counts by year.	Tumour-based pathology counts; multiple tumours could contribute. In situ status was specifically described for melanoma, not as a principal BCC/SCC outcome.	Mean crude incidence 208.97/100,000 for BCC and 75.15/100,000 for SCC; BCC +47% and SCC +80% from 2014 to 2023; 2020 diagnostic dip.	Mortality not evaluated.	Single regional hospital/pathology catchment; crude BCC/SCC rates and local referral patterns limit national generalisability.
15. Kappelin et al., Acta Derm Venereol. 2024;104:adv40112. doi:10.2340/actadv.v104.40112	Sweden<br>Study period: 2004-2017	Nationwide registry-based cohort study<br>Population: All individuals with histopathologically verified primary BCCs in the Swedish BCC Registry<br>Data source: Swedish BCC Registry	Histopathologically verified primary BCC.	Multiple primary BCCs explicitly included and modelled; 39.9% of affected individuals had at least two registered BCCs. In situ disease not applicable to BCC endpoint.	Five-year risk of another BCC after the first diagnosis ~30% in men and 27% in women; risk increased after multiple prior BCCs.	Mortality not evaluated.	Focus on multiplicity rather than population incidence trend; findings depend on completeness of histopathology-based BCC registration.
16. Hunter & Haylock, Int J Cancer. 2026;158:574-586. doi:10.1002/ijc.70096	United Kingdom<br>Study period: 1955-2011 follow-up; publication online 2025 / issue 2026	Occupational cohort study<br>Population: 172,452 UK radiation workers; >5.3 million person-years<br>Data source: UK National Registry for Radiation Workers linked to cancer registration	First primary BCC and SCC; 5,748 NMSC cases (4,288 BCC; 818 SCC reported in principal subtype analyses).	First-primary cancer analysis; anatomical site used as proxy for UV exposure; in situ disease not a principal endpoint.	Radiation dose-response observed for BCC before adjustment for anatomical site; association attenuated after UV-proxy adjustment. SCC association weak.	Study focused on incidence rather than NMSC mortality.	Occupational cohort; residual confounding by individual UV exposure; historical exposure assessment and first-primary approach limit generalisability.
17. Zhou et al., Scientific Reports. 2025;15:5996. doi:10.1038/s41598-025-90485-3	Global, with European regional and national estimates<br>Study period: 1990-2021	Global burden/trend analysis<br>Population: GBD-covered populations, including Europe<br>Data source: Global Burden of Disease study estimates	Melanoma and NMSC burden, including BCC and SCC components where available.	Model-based population estimates; not a tumour-level registry study; in situ disease not separately counted.	Increasing absolute NMSC burden and age-related concentration of disease; regional/national temporal trends reported using age-standardised metrics.	NMSC mortality and burden metrics assessed at global/regional/national level.	Modelled estimates inherit heterogeneity and under-registration from source data; BCC reporting is incomplete in many countries.
18. Balkenhol et al., Eur J Cancer. 2025;230:116068. doi:10.1016/j.ejca.2025.116068	Germany<br>Study period: 1986-2019	Population-based registry analysis<br>Population: Patients with registered cSCC in German cancer-registry data<br>Data source: German population cancer registry data	cSCC analysed by incidence, anatomical site, stage and histological subtype.	Histology-specific invasive cSCC registration; multiple-primary handling followed registry rules; in situ lesions were outside the principal invasive cSCC endpoint.	Crude cSCC incidence increased by >500% from 1986 to 2015. The authors reported a subsequent plateau, except for a delayed increase in Saarland. This percentage change is not an age-standardized trend.	Mortality was not the central endpoint of this source.	Historical changes in registry coverage/coding and tumour-counting rules may influence long-term trends.

Reference	Country/region & period	Design; population; data source	Case definition / ascertainment	Multiple tumours / in situ	Key quantitative epidemiological results	Mortality / survival	Main methodological limitations
19. Zwierko & Sulkowska. Registration and Epidemiology of Malignant Neoplasms in Warsaw, 1963-2013. Mazovian Cancer Registry, 2016	Warsaw/Mazovia, Poland<br>Study period: 1963-2013	Long-term population cancer-registry report<br>Population: Residents covered by the Warsaw/Mazovian Cancer Registry<br>Data source: Mazovian Cancer Registry	Malignant neoplasms by ICD site, including C44 malignant neoplasms of skin; BCC/cSCC are not separated.	Person/registry-based C44 reporting according to registry rules; multiple keratinocyte tumours and in situ lesions are not represented as histology-specific outcomes.	Provides historical C44 case counts and incidence/mortality context for the Warsaw/Mazovian population.	Cancer-site mortality tables are included at registry level, but BCC/cSCC-specific mortality is unavailable.	Historical contextual source ending in 2013; aggregated C44 prevents histology-specific interpretation and limits relevance to recent trends.
22. Ferlay et al., Global Cancer Observatory: Cancer Today. IARC, 2024	Europe and individual European countries<br>Study period: Estimates for 2022 (platform accessed 31 March 2026)	International cancer surveillance database/report<br>Population: National populations represented in GLOBOCAN<br>Data source: IARC Global Cancer Observatory / GLOBOCAN	Modelled NMSC category as defined in the selected GLOBOCAN output; it cannot automatically be equated with combined BCC and cSCC incidence.	Country estimates depend on source registries and modelling; BCC is incompletely registered in many systems; in situ disease not part of standard malignant cancer incidence estimates.	Cross-sectional country-level burden estimates. No numerical GLOBOCAN total or temporal trend is used in the comparative table.	Country-level mortality estimates available for the defined NMSC category.	Not a harmonised histology-specific BCC/cSCC registry; estimates depend on availability/quality of national source data.
23. Garbe et al., J Dtsch Dermatol Ges. 2023;21 Suppl 5:3-11. doi:10.1111/ddg.15170	Germany<br>Study period: 2010-2015	Retrospective population-based secondary-data study using a 40% anonymized sample of DAK statutory health-insurance data (2,319,584 insured persons; closed screening cohort 1,290,725 aged >=35 years).	Skin-cancer diagnoses identified from ICD-10 codes C43/C44/D03/D04 and linked to screening/excision claims; C44/D04 capture non-melanocytic malignant/in-situ skin tumours.	Incident diagnoses required an 8-quarter diagnosis-free period. Analysis was patient-based rather than tumour-based; in situ lesions were explicitly included through D03/D04.	Biennial screening participation in 2014-2015: 33.6% women and 32.6% men. A skin-cancer finding occurred in 4.2% of screened persons; 50.1% of incident diagnoses were detected through screening; 1.5% developed an interval tumour within two years after a negative screen.	Mortality was not assessed.	Claims-based ascertainment; melanoma and NMSC are partly aggregated in screening metrics; pathology/histology detail and multiple-primary counting are limited.
24. Navarro-Bielsa et al., Eur J Dermatol. 2022;32:724-730. PMID:36856390	Spain<br>Cross-sectional occupational study	Occupational dermatologic prevalence study among 215 farm workers (84.4% men; mean age 65.8 years).	Dermatologic assessment of actinic keratosis, BCC and cSCC; BCC and cSCC prevalence reported separately.	Multiplicity and in situ counting were not the principal study endpoints.	Actinic keratosis: 41.4%; BCC: 9.8%; cSCC: 1.4%. The study documented a high burden of UV-related keratinocyte lesions in a chronically exposed occupational group.	Mortality was not assessed.	Small occupational sample, cross-sectional design and limited generalizability to the general Spanish population; prevalence rather than population incidence.
25. Cozzi et al., J Plast Reconstr Aesthet Surg. 2022;75:3616-3621. doi:10.1016/j.bjps.2022.06.037	Northern Italy (Bergamo and Varese)<br>Comparison: Dec 2019-Jan 2020 vs Dec 2020-Jan 2021	Retrospective multicentre real-world surgical/pathology study; 214 patients and 274 excised NMSC lesions.	Consecutive histopathology reports were screened for BCC or cSCC; clinical and surgical waiting-time data were linked.	Lesion-based analysis allowed multiple tumours per patient; only invasive BCC/cSCC selected, with no separate in-situ category.	cSCC proportion increased from 27.4% to 44.5%; BCC decreased from 72.6% to 55.5%. Mean tumour diameter increased from 13.3 mm to 22.7 mm after the pandemic disruption; cSCC and BCC diameters both increased significantly.	Mortality was not assessed.	Two short time windows, surgical case selection and no population denominator; findings reflect care prioritization and case-mix rather than true incidence.
26. Basic-Jukic et al., Croat Med J. 2022;63:148-155. doi:10.3325/cmj.2022.63.148	Croatia<br>Approximately 40-year transplant cohort (1974-2014)	Retrospective cohort of 1,232 renal transplant recipients treated at University Hospital Center Zagreb.	NMSC occurrence analysed with BCC and cSCC reported separately in transplant recipients.	5.28% developed one NMSC and the remainder of affected patients had multiple NMSCs; 8.3% of affected patients developed both BCC and cSCC. In situ disease was not the target outcome.	NMSC prevalence 6.81% (84/1,232). Among affected patients, 60.7% developed BCC, 30.9% cSCC and 8.3% both; BCC:cSCC ratio 1.76:1. Risk was higher in men and older transplant recipients.	Mortality was not the principal outcome.	Single-centre transplant population, retrospective design and nonuniform dermatologic surveillance; not directly generalizable to the general population.
27. Zlatarova & Dokova, Ophthalmic Epidemiol. 2021;28:198-204. doi:10.1080/09286586.2020.1808230	Bulgaria<br>Study period: 2000-2015	National cancer-registry incidence study using all cases coded ICD-10 C44.1; national and Varna-region estimates.	Non-melanoma eyelid malignancies identified from the Bulgarian National Cancer Registry; crude and directly age-standardized incidence rates calculated.	Registry rules for multiple tumours were not detailed in the report; in situ lesions were excluded by use of malignant C44.1 coding.	3,046 cases. ASR per 100,000: men 2.9 (95% CI 2.79-3.11), women 2.4 (2.30-2.54), both sexes 2.6 (2.53-2.73), standardized to the 2013 European Standard Population. ASR increased in women and both sexes combined.	Mortality was not assessed.	Site-specific eyelid disease only; C44.1 does not distinguish BCC from cSCC histology; registry multiple-primary rules not fully described.
28. Cayuela et al., Eur J Dermatol. 2024;34:392-397. doi:10.1684/ejd.2024.4732	Spain<br>Study period: 1990-2019	Nationwide ecological trend analysis using GHDx/GBD model-based estimates for the Spanish population.	NMSC analysed as an aggregate keratinocyte-carcinoma burden; age-standardized incidence rates calculated and Joinpoint plus age-period-cohort analyses performed.	Model-based population estimates; tumour multiplicity and in situ lesions were not separately counted at the patient/tumour level.	2,302,399 NMSC cases estimated for 1990-2019. ASIRs declined after 2005; men had slightly higher rates. Joinpoint and age-period-cohort analyses showed sex- and cohort-specific trend differences.	Mortality was not the principal outcome of this incidence-trend analysis.	GBD/GHDx model-based estimates depend on source-data completeness; BCC and cSCC are not fully separated and registry-specific multiple-primary rules cannot be evaluated.

Reference	Country/region & period	Design; population; data source	Case definition / ascertainment	Multiple tumours / in situ	Key quantitative epidemiological results	Mortality / survival	Main methodological limitations
29. Mangone et al., Ann Res Oncol. 2022;2:105-115. doi:10.48286/aro.2022.41	Reggio Emilia, Northern Italy<br>Study period: 2001-2020; direct pandemic comparison 2019 vs 2020	Population-based cancer-registry time-trend/pandemic analysis<br>Population: Residents covered by Reggio Emilia Cancer Registry<br>Data source: Reggio Emilia Cancer Registry	All cancers by site; non-melanoma skin cancer reported as a separate skin site category for pandemic comparison.	Registry case counts; histology-specific BCC/cSCC and multiple-primary rules were not detailed for the NMSC subgroup.	In 2020, 4,031 cancers were recorded, 669 fewer than 2019 (-14.2%); non-melanoma skin cancer showed the largest absolute decline (-281 cases).	Overall cancer incidence/mortality trends reported; NMSC-specific mortality not separately quantified.	NMSC reported as an aggregated category; pandemic-related fall reflects diagnostic activity as well as possible true temporal variation.
30. Makaranka et al., Cureus. 2022;14:e25019. doi:10.7759/cureus.25019	Scotland and North Cancer Alliance, UK<br>Study period: 2019-2020	Population registry before-after pandemic comparison<br>Population: Scottish population; NCA catchment ~1.4 million<br>Data source: Public Health Scotland cancer dashboard / Scottish Cancer Registration Team	Pathologically confirmed non-melanoma skin cancer as defined in Scottish registry outputs.	Registry diagnosis counts; histology-specific BCC/cSCC and multiple-primary rules were not separately reported in the paper.	Recorded NMSC diagnoses were lower in 2020 than in 2019. The source gives Scottish counts of 21,626 and 16,193 but reports a 23.3% reduction, which is arithmetically inconsistent with those counts. The synthesis therefore reports the direction without reproducing a percentage reduction.	Mortality not evaluated.	Ecological before-after comparison; cannot distinguish delayed diagnosis from true incidence change; aggregate NMSC category.

The table describes all 24 included sources; contextual references are not included in this inventory. Publication eligibility dates and observation periods are distinct. The first-column number is the manuscript reference number and links directly to the corresponding row in Supplementary Table S2B; it is not a sequential row count.
