# Supplementary material for: Epidemiology of Non-Melanoma Skin Cancer in Europe over the Last Decade: A Systematic Review
Source: Cancers (Basel). 2026 Sep 14;18(18):2970. doi: 10.3390/cancers18182970 (PMC13605927; doi:10.3390/cancers18182970)
Supplement: Supplementary file 1 [file cancers-18-02970-s001.zip › Supplementary_Table_S2B.pdf]

Supplementary Table S2B. Descriptive Source-Level Methodological Appraisal of the 24 Included Sources

Appraisal approach. Each source is described across nine domains. L = low methodological concern; S = some methodological concern; H = high methodological concern. These descriptive judgments support the intended use of each source in the synthesis; they are not a validated risk-of-bias tool, numerical score, or formal certainty grading. Overall methodological concern reflects the pattern of limitations and intended use, not an arithmetic sum or automatic worst-domain rule. No source was excluded solely because of its appraisal. Reference numbers match the manuscript and Supplementary Table S2. The row set contains exactly 24 sources; gaps in reference numbering denote contextual references outside the included set.

Domains: D1 population coverage; D2 case ascertainment; D3 histopathological confirmation; D4 reporting completeness; D5 BCC/cSCC distinction; D6 multiple primary tumours; D7 rate standardization; D8 observation duration; D9 statistical transparency.

Reference	D1	D2	D3	D4	D5	D6	D7	D8	D9	Overall	Brief rationale
1. Ciążyńska et al. (2021)	S	S	L	S	L	S	S	L	S	S	Multicentre clinical/pathology series plus NHF context; not a complete population registry and national C44 data aggregate histologies.
2. Sendín-Martín et al. (2026)	S	S	H	S	S	H	L	L	L	S	Large European GBD analysis with strong temporal/statistical methods, but model-based rather than tumour-level pathology data and limited multiple-primary information.
3. Keim et al. (2023)	L	L	L	S	L	S	L	L	L	L	Population registries with histology-specific cSCC ascertainment and transparent trend methods; residual cross-registry differences remain.
8. Wysocki et al. (2025)	L	S	S	S	L	H	S	L	S	S	Nationwide claims cohort with broad coverage, but tumour-level pathology and multiple-primary detail are limited in administrative data.
9. Bartoš & Kullová (2018)	H	L	L	S	L	L	H	S	S	S	Histologically verified clinicopathological series, but single-centre referral population and no population denominator or standardized incidence.
10. Krogh Holdam et al. (2025)	L	L	L	S	L	H	L	L	L	L	Nationwide population registers with strong case ascertainment and standardization; first-incidence design underestimates total multiple-tumour workload.
11. Duffau et al. (2026)	L	L	L	L	L	S	L	L	L	L	Nationwide pathology/cancer registry cohort with histological confirmation and explicit invasive/in-situ separation; subsequent primaries not fully quantified.
12. Callens et al. (2016)	L	L	L	S	L	L	L	L	L	L	National pathology-driven registry with multiple tumours captured; registration completeness evolved over time but methods and rates are transparent.
13. Fernández-Martínez et al. (2026)	S	L	L	L	L	H	L	L	L	L	Multicentre population registries with histologically defined first invasive cSCC and standardized rates; incomplete national coverage and first-tumour approach.

Reference	D1	D2	D3	D4	D5	D6	D7	D8	D9	Overall	Brief rationale
14. Santos-Juanes et al. (2025)	H	L	L	S	L	L	H	S	S	S	Regional pathology registry with strong histological ascertainment but limited population coverage and crude, rather than standardized, BCC/SCC rates.
15. Kappelin et al. (2024)	L	L	L	L	L	L	S	L	L	L	Nationwide histopathology-based BCC registry with explicit multiplicity modelling; study focuses on subsequent BCC risk rather than national incidence trend.
16. Hunter & Haylock (2026)	S	L	L	S	L	H	S	L	L	S	Large linked occupational cohort with cancer registration, but first-primary approach and residual UV confounding limit generalisability.
17. Zhou et al. (2025)	S	S	H	S	S	H	L	L	L	S	GBD-based global/regional modelling with standardized metrics; estimates inherit source-data under-registration and are not tumour-level pathology records.
18. Balkenhol et al. (2025)	L	L	L	S	L	S	L	L	L	L	Population registry analysis with histology-specific invasive cSCC and transparent long-term methods; historical coding/counting changes remain possible.
19. Zwierko & Sulkowska (2016)	S	S	H	S	H	H	S	L	S	S	Historical C44 registry report useful for context, but histology is aggregated and multiple tumours/in-situ disease are not separable.
22. Ferlay et al. / GLOBOCAN (2024)	S	S	H	S	H	H	L	S	L	S	International modelled surveillance estimates with broad coverage and standardized rates; limited histology-specific BCC/cSCC and multiple-primary ascertainment.
23. Garbe et al. (2023)	L	S	S	S	H	H	S	L	S	S	Claims-based national screening evaluation; useful population coverage but case definition, histology and multiple-primary detail are less complete than pathology registries.
24. Navarro-Bielsa et al. (2022)	H	L	L	S	L	S	H	H	S	S	Small cross-sectional occupational sample with histological confirmation; no population incidence denominator and limited generalisability.
25. Cozzi et al. (2022)	H	L	L	S	L	L	H	H	S	S	Multicentre surgical/pathology series with confirmed BCC/cSCC, but selected surgical cases, short time windows and no population denominator.
26. Bašić-Jukić et al. (2022)	H	L	L	S	L	L	H	L	S	S	Single-centre transplant cohort with confirmed NMSC subtype data; high-risk selected population and nonuniform surveillance limit population inference.
27. Zlatarova & Dokova (2021)	L	S	S	S	H	S	L	L	L	S	National cancer-registry incidence study with standardized rates, but ICD-10 C44.1 does not provide full BCC/cSCC histology separation.
28. Cayuela et al. (2024)	S	S	H	S	S	H	L	L	L	S	Nationwide Spanish GBD-based trend analysis with standardized methods; model dependence and limited tumour-level histology/multiplicity remain.

Reference	D1	D2	D3	D4	D5	D6	D7	D8	D9	Overall	Brief rationale
29. Mangone et al. (2022)	L	S	S	S	H	S	S	L	S	S	Population cancer registry study of pandemic-period diagnoses; NMSC is aggregated and histology/multiple-primary rules are incompletely detailed.
30. Makaranka et al. (2022)	L	L	L	S	H	S	S	S	S	S	Population-based Scottish diagnostic comparison with pathology-confirmed registry diagnoses; short observation window and aggregate NMSC reporting constrain inference.

The five authors jointly undertook this descriptive appraisal. Domain ratings describe limitations for the intended interpretation of a source; they do not establish a validated risk-of-bias classification.
