# Supplementary material for: Epidemiology of Non-Melanoma Skin Cancer in Europe over the Last Decade: A Systematic Review
Source: Cancers (Basel). 2026 Sep 14;18(18):2970. doi: 10.3390/cancers18182970 (PMC13605927; doi:10.3390/cancers18182970)
Supplement: Supplementary file 1 [file cancers-18-02970-s001.zip › Supplementary_Table_S3.pdf]

Supplementary Table S3. Full-text reports excluded after eligibility assessment

One principal reason per unique excluded report (37 rows). The first column gives row number / retained candidate identifier; neither is a manuscript reference number.

Eligibility was assessed against the review question and the criteria stated in Methods. Reports in which NMSC appeared only as an incidental etiologic/safety endpoint without an eligible NMSC burden, incidence/prevalence, temporal-trend, mortality, registration, or pandemic-related diagnostic/care outcome were excluded. The exact duplicate identified during reconciliation was moved to the pre-screening duplicate-removal step and is therefore not listed below. Candidate numbers are retained as source identifiers and are not manuscript reference numbers.

Row / candidate ID	Reference	Principal reason for exclusion
1 / 1	Autio E, Vahlberg T, Ekman E, Laaksonen I, Hemmilä M, Eskelinen A, Mäkelä K. Long-term cancer risk after metal-on-metal hip arthroplasty: a Finnish population-based study with a mean follow-up of 12.8 years. BMJ Open. 2026 Aug 19;16(8):e114178. doi: 10.1136/bmjopen-2025-114178. PMID: 42624603.	Publication outside the prespecified search period (published 19 August 2026; cutoff 31 March 2026).
2 / 2	Garbe C, Keim U, Amaral T, Ascierto PA, Bauer J, Dummer R, Eggermont AMM, Eigentler TK, Flatz L, Forchhammer S, Forschner A, Gandini S, Gershenwald JE, Hauschild A, Hoeller C, Kirkwood JM, Lebbe C, Long GV, Lorigan P, Luke JJ, Meier F, Metzler G, Nathan P, Postow MA, Robert C, Röcken M, Schadendorf D, Weide B, Whiteman D, Leiter U, Martus P. Years of life lost in patients with a false-negative diagnosis of primary melanoma. A prospective study of the German Central Malignant Melanoma Registry involving 9063 patients over 28 years. Nat Commun. 2026 Jul 2;17(1):5790. doi: 10.1038/s41467-026-74443-9. PMID: 42393046; PMCID: PMC13328647.	Melanoma-only study; no eligible NMSC/BCC/cSCC outcome.
3 / 3	Gambichler T, Heinzer E, Abu Rached N, Schulze HJ, Noah K, Hofmann SC, Wesselmann U, Gutzmer R, Boms S, Susok L, Weyer-Fahlbusch SS, Kreuter A, Hyun J, Müller VL, Auer R, Becker JC. General health versus tumor stage: determinants of survival in Merkel cell carcinoma assessed by sentinel lymph node biopsy. J Cancer Res Clin Oncol. 2026 Apr 28;152(4):100. doi: 10.1007/s00432-026-06485-x. PMID: 42050004; PMCID: PMC13125591.	Merkel cell carcinoma study; outside the operational review condition of keratinocyte carcinoma (BCC/cSCC/NMSC).
4 / 5	Brandtzæg KH, von der Lippe C, Vestengen IK, Holtan JP, Winge MI, Rustad CF, Bergersen TK, Sigurdardottir S, Sjursen W, Gjersvik P, Hortemo KH. Clinical and genetic findings in 103 individuals in Norway with basal cell naevus syndrome. Br J Dermatol. 2026 Jan 27;194(2):264-272. doi: 10.1093/bjd/ljaf413. PMID: 41129277.	Clinical-genetic basal cell naevus syndrome cohort; does not provide an eligible population-burden, temporal-trend, registry, mortality, or pandemic-disruption endpoint.
5 / 6	Wehner MR. Keratinocyte Carcinoma: A Review. JAMA. 2026 Jan 6;335(1):70-81. doi: 10.1001/jama.2025.18749. PMID: 41165676.	Narrative clinical review; not an eligible primary epidemiological study, registry report, or systematic review with extractable study-level epidemiological data.
6 / 7	Beraia A, Znaor A, Anda EE, Gamkrelidze A, Borch KB. Incidence of common cancers in Georgia in 2015-2019: A national cancer registry study. Cancer Treat Res Commun. 2026;48:101267. doi: 10.1016/j.ctarc.2026.101267. Epub 2026 May 29. PMID: 42258958.	Publication outside the prespecified search period (published after 31 March 2026).
7 / 8	Lee YH, Song GG. Cancer risk across sex, region, and disease subtype in ANCA-associated vasculitis patients : A meta-analysis. Z Rheumatol. 2025 Dec;84(Suppl 5):210-217. English. doi: 10.1007/s00393-025-01717-w. Epub 2025 Sep 1. PMID: 40890556.	Mixed-region cancer-risk meta-analysis; the NMSC estimate is an etiologic risk endpoint and is not separately reported as a European NMSC burden/trend estimate.
8 / 9	Petrova D, Sánchez-Martín A, Redondo-Sánchez D, Madrid Pérez-Esparza B, Suárez Betancourt L, González-Flores E, Mendoza-García O, Jiménez-Moleón JJ, Sánchez MJ. What cancers are increasing in young adults: a population-based study of incidence trends in adults 20-49 years old in the South of Spain. BMC Public Health. 2025 Nov 22;26(1):16. doi: 10.1186/s12889-025-25707-4. PMID: 41275217; PMCID: PMC12764024.	All-cancer incidence study in young adults; no separately reported eligible NMSC/BCC/cSCC epidemiological outcome.

Row / candidate ID	Reference	Principal reason for exclusion
9 / 11	Chanal J. Prise en charge du carcinome basocellulaire en 2025 [Management of basal cell carcinoma in 2025]. Ann Chir Plast Esthet. 2025 Nov;70(6):452-456. French. doi: 10.1016/j.anplas.2025.05.009. PMID: 41232966.	Clinical management review of BCC; no eligible quantitative epidemiological burden/trend/registry outcome.
10 / 12	Morel J, Wetzman A, Wendling D, Soubrier M, Hoang S, Briançon D, Roth O, Goupille P, Gottenberg JE, Mariette X, Lukas C; French Society of Rheumatology, the investigators participating in REGATE registry. Risk of cancer in patients with rheumatoid arthritis under tocilizumab: Data from the French national registry REGATE. Joint Bone Spine. 2025 Oct;92(5):105900. doi: 10.1016/j.jbspin.2025.105900. Epub 2025 Apr 4. PMID: 40189190.	Rheumatoid-arthritis treatment-safety registry; NMSC is an incidental secondary cancer outcome rather than a NMSC-focused burden/trend/registration endpoint.
11 / 13	Langselius O, Rumgay H, de Vries E, Whiteman DC, Jemal A, Parkin DM, Soerjomataram I. Global burden of cutaneous melanoma incidence attributable to ultraviolet radiation in 2022. Int J Cancer. 2025 Sep 15;157(6):1110-1119. doi: 10.1002/ijc.35463. Epub 2025 May 27. PMID: 40421619.	Melanoma-only burden study.
12 / 14	Perrier F, Ahimbisibwe A, Ghiasvand R, Rueegg CS, Green AC, Borch KB, Braaten T, Weiderpass E, Valberg M, Robsahm TE, Veierød MB. Physical activity and mortality in melanoma patients within the Norwegian Women and Cancer study (NOWAC). Int J Cancer. 2025 Sep 1;157(5):846-857. doi: 10.1002/ijc.35430. Epub 2025 Apr 3. PMID: 40181554.	Melanoma-only mortality study.
13 / 15	Angelini M, Seyyedsalehi MS, Boffetta P. Occupational benzene exposure and skin cancers: a systematic review and meta-analysis. Occup Med (Lond). 2025 Jul 14;75(3):163-170. doi: 10.1093/occmed/kqae112. PMID: 40036809; PMCID: PMC12257946.	Exposure-focused etiologic meta-analysis; reports relative risks for benzene exposure rather than a European population NMSC burden/trend/registration estimate.
14 / 16	Jonušienė G, Kačėnienė A, Gedvilaitė V, Dadonienė J, Miltinienė D, Deresevičienė G, Smailytė G. Cancer risk in patients with Sjögren's syndrome in Lithuanian cohort study. Clin Rheumatol. 2025 Jul;44(7):2883-2887. doi: 10.1007/s10067-025-07489-7. Epub 2025 May 19. PMID: 40383746.	Disease-specific cancer-risk cohort; NMSC is a secondary SIR outcome rather than a NMSC-focused burden/trend/registration endpoint.
15 / 18	Wang R, Xu Y, Zhang Y, Wu Y, Zuo A, Liu S, Ba Y, Xu H, Weng S, Zhou Z, Ma H, Luo P, Cheng Q, Han X, Liu Z. Total and regional fat-to-muscle mass ratio and risks of pan-cancer: a prospective cohort study. BMC Med. 2025 May 28;23(1):296. doi: 10.1186/s12916-025-04102-1. PMID: 40437455; PMCID: PMC12121253.	Pan-cancer etiologic cohort; NMSC is one secondary site-specific association and the study does not characterize NMSC burden/trends/registration.
16 / 19	Chen W, Pang L, Wei X, Lan Y, Su X, Dong Y, Zhu Z, Bai J, Zhou J, Cui H, Zhang B. Micronutrients and skin cancer risk: a Mendelian randomization study. World J Surg Oncol. 2025 May 11;23(1):180. doi: 10.1186/s12957-025-03814-1. PMID: 40350422; PMCID: PMC12067739.	Mendelian-randomization etiologic study; evaluates genetically proxied micronutrient effects rather than descriptive NMSC epidemiology, registration, mortality, or diagnostic disruption.
17 / 20	Wewer MD, Letnar G, Andersen KK, Malham M, Wewer V, Seidelin JB, Bendtsen F, Burisch J. Thiopurines and the Risk of Cancer in Patients With Inflammatory Bowel Disease and Reference Individuals Without Inflammatory Bowel Disease: A Danish Nationwide Cohort Study (1996-2018). Clin Gastroenterol Hepatol. 2025 May;23(6):1030-1038. doi: 10.1016/j.cgh.2024.08.006. Epub 2024 Aug 28. PMID: 39209201.	IBD treatment-exposure cancer-risk study; NMSC is a secondary safety outcome rather than a NMSC-focused epidemiological burden/trend source.
18 / 21	Tasdogan A, Sullivan RJ, Katalinic A, Lebbe C, Whitaker D, Puig S, van de Poll-Franse LV, Massi D, Schadendorf D. Cutaneous melanoma. Nat Rev Dis Primers. 2025 Apr 3;11(1):23. doi: 10.1038/s41572-025-00603-8. PMID: 40180935.	Melanoma review; no eligible NMSC/BCC/cSCC outcome.
19 / 23	Garbe C, Amaral T, Peris K, Hauschild A, Arenberger P, Basset-Seguin N, Bastholt L, Bataille V, Brochez L, Del Marmol V, Dréno B, Eggermont AMM, Fargnoli MC, Forsea AM, Höller C, Kaufmann R, Kelleners-Smeets N, Lallas A, Lebbé C, Leiter U, Longo C, Malvehy J, Moreno-Ramirez D, Nathan P, Pellacani G, Saiag P, Stockfleth E, Stratigos AJ, Van Akkooi ACJ, Vieira R, Zalaudek I, Lorigan P, Mandala M; European Association of Dermato-Oncology (EADO), the European Dermatology Forum (EDF), and the European Organization for Research and Treatment of Cancer (EORTC). European consensus-based interdisciplinary guideline for melanoma. Part 2: Treatment - Update 2024. Eur J Cancer. 2025 Jan 17;215:115153. doi: 10.1016/j.ejca.2024.115153. Epub 2024 Nov 29. PMID: 39709737.	Melanoma treatment guideline; wrong condition and no eligible NMSC epidemiological outcome.

Row / candidate ID	Reference	Principal reason for exclusion
20 / 24	Garbe C, Amaral T, Peris K, Hauschild A, Arenberger P, Basset-Seguin N, Bastholt L, Bataille V, Brochez L, Del Marmol V, Dréno B, Eggermont AMM, Fargnoli MC, Forsea AM, Höller C, Kaufmann R, Kelleners-Smeets N, Lallas A, Lebbé C, Leiter U, Longo C, Malvehy J, Moreno-Ramirez D, Nathan P, Pellacani G, Saiag P, Stockfleth E, Stratigos AJ, Van Akkooi ACJ, Vieira R, Zalaudek I, Lorigan P, Mandala M; European Association of Dermato-Oncology (EADO), the European Dermatology Forum (EDF), and the European Organization for Research and Treatment of Cancer (EORTC). European consensus-based interdisciplinary guideline for melanoma. Part 1: Diagnostics - Update 2024. Eur J Cancer. 2025 Jan 17;215:115152. doi: 10.1016/j.ejca.2024.115152. Epub 2024 Nov 28. PMID: 39700658.	Melanoma diagnostic guideline; wrong condition and no eligible NMSC epidemiological outcome.
21 / 25	Tschopp S, Ciernik A, Dummer R, Imhof L. X-Ray Therapy of Skin Cancer at Critical Facial Sites: Results of a Tertiary Referral Center. Dermatology. 2025;241(5-6):499-510. doi: 10.1159/000548154. Epub 2025 Aug 26. PMID: 40875679; PMCID: PMC12688331.	Treatment-outcome series of radiotherapy for skin cancer; no population epidemiological burden/trend/registry endpoint.
22 / 26	Pedersen JE, Petersen KU, Andersen MG, Saber AT, Vogel U, Ebbehøj NE, Jensen TK, Wils RS, Bonde JP, Hansen J. Cancer incidence in a cohort of Danish firefighters: An extended long-term follow-up 1968-2021. Am J Ind Med. 2024 Sep;67(9):857-864. doi: 10.1002/ajim.23635. Epub 2024 Jul 4. PMID: 38965801.	Occupational cancer cohort reports melanoma, not an eligible NMSC/BCC/cSCC endpoint.
23 / 30	Johansson ALV, Kønig SM, Larønningen S, Engholm G, Kroman N, Seppä K, Malila N, Steig BÁ, Gudmundsdóttir EM, Ólafsdóttir EJ, Lundberg FE, Andersson TM, Lambert PC, Lambe M, Pettersson D, Aagnes B, Friis S, Storm H. Have the recent advancements in cancer therapy and survival benefitted patients of all age groups across the Nordic countries? NORDCAN survival analyses 2002-2021. Acta Oncol. 2024 Apr 10;63:179-191. doi: 10.2340/1651-226X.2024.35094. PMID: 38597666; PMCID: PMC11332520.	All-cancer Nordic survival analysis; no separately reported eligible NMSC/BCC/cSCC outcome.
24 / 31	Michailovich YI, Sumkina OV, Gorokh YL. CANCER INCIDENCE IN UKRAINE: TRENDS IN 2010-2019 AND THE IMPACT OF COVID-19 PANDEMIC. Exp Oncol. 2024 Feb 3;45(4):463-473. doi: 10.15407/exp-oncology.2023.04.463. PMID: 38328843.	All-cancer Ukrainian incidence/COVID study; no separately extractable NMSC/BCC/cSCC quantitative outcome.
25 / 33	Queirolo P, Cinquini M, Argenziano G, Bassetto F, Bossi P, Boutros A, Clemente C, de Giorgi V, Del Vecchio M, Patuzzo R, Peris K, Quaglino P, Reali A, Zalaudek I, Spagnolo F. Guidelines for the diagnosis and treatment of basal cell carcinoma: a GRADE approach for evidence evaluation and recommendations by the Italian Association of Medical Oncology. ESMO Open. 2023 Dec;8(6):102037. doi: 10.1016/j.esmoop.2023.102037. Epub 2023 Oct 23. PMID: 37879235; PMCID: PMC10598491.	Clinical BCC guideline; no original eligible epidemiological dataset.
26 / 34	Lalloo F, Kulkarni A, Chau C, Nielsen M, Sheaff M, Steele J, van Doorn R, Wadt K, Hamill M, Torr B, Tischkowitz M; Delphi respondents; Hanson H. Clinical practice guidelines for the diagnosis and surveillance of BAP1 tumour predisposition syndrome. Eur J Hum Genet. 2023 Nov;31(11):1261-1269. doi: 10.1038/s41431-023-01448-z. Epub 2023 Aug 22. PMID: 37607989; PMCID: PMC10620132.	Clinical practice guideline for BAP1 tumour predisposition syndrome; no eligible NMSC epidemiological dataset.
27 / 35	Trächsel B, Rousson V, Bulliard JL, Locatelli I. Comparison of statistical models to predict age-standardized cancer incidence in Switzerland. Biom J. 2023 Oct;65(7):e2200046. doi: 10.1002/bimj.202200046. Epub 2023 Apr 20. PMID: 37078835.	Methodological cancer-prediction paper; does not report a separate NMSC/BCC/cSCC epidemiological dataset.
28 / 36	Lewandowska A, Lewandowski T, Rudzki G, Próchnicki M, Laskowska B, Pavlov S, Vlasenko O, Rudzki S, Wójcik W. The Risk of Melanoma due to Exposure to Sun and Solarium Use in Poland: A Large-Scale, Hospital Based Case - Control Study. Asian Pac J Cancer Prev. 2023 Jul 1;24(7):2259-2269. doi: 10.31557/APJCP.2023.24.7.2259. PMID: 37505755; PMCID: PMC10676492.	Melanoma-only case-control study.
29 / 37	Kristiansen MF, Mikkelsen RM, Kristiansdóttir T, Andórsdóttir G, Hansen SÓ, Á Steig B, Nielsen KR, Skaalum Petersen M, Strøm M. Cancer survival in the Faroe Islands over the last 50 years compared to the other Nordic countries. Int J Cancer. 2023 May 15;152(10):2090-2098. doi: 10.1002/ijc.34456. Epub 2023 Feb 13. PMID: 36727543.	All-cancer survival study; no separately reported NMSC/BCC/cSCC outcome.
30 / 38	Bician P, Mego M. Ethnic differences in the incidence of childhood cancer. Klin Onkol. 2023 Spring;36(3):192-197. English. doi: 10.48095/ccko2023192. PMID: 37353347.	Childhood cancer study; no eligible NMSC/BCC/cSCC outcome.

Row / candidate ID	Reference	Principal reason for exclusion
31 / 39	Trama A, Stark D, Bozovic-Spasojevic I, Gaspar N, Peccatori F, Toss A, Bernasconi A, Quarello P, Scheinemann K, Jezdic S, Blondeel A, Mountzios G, Bielack S, Saloustros E, Ferrari A. Cancer burden in adolescents and young adults in Europe. ESMO Open. 2023 Feb;8(1):100744. doi: 10.1016/j.esmoop.2022.100744. Epub 2023 Feb 6. PMID: 36753992; PMCID: PMC10024081.	Adolescent/young-adult all-cancer burden study; no separately reported NMSC/BCC/cSCC outcome.
32 / 40	Kalashnykova NM. CHARACTERISTICS OF THE VOLUME AND OUTCOMES OF MEDICAL CARE FOR PATIENTS WITH SKIN CANCER IN UKRAINE IN 2010-2020. Wiad Lek. 2023;76(4):799-804. doi: 10.36740/WLek202304115. PMID: 37226618.	Skin-cancer health-services study, but NMSC-specific quantitative outcomes are not separately extractable from the reported results.
33 / 43	Kuiper JG, Overbeek JA, Foch C, Boutmy E, Sabidó M. Incidence of malignancies in patients with multiple sclerosis versus a healthy matched cohort: A population-based cohort study in the Netherlands using the PHARMO Database Network. J Clin Neurosci. 2022 Sep;103:49-55. doi: 10.1016/j.jocn.2022.06.025. Epub 2022 Jul 8. PMID: 35810606.	Multiple-sclerosis cancer-risk cohort; NMSC is not a NMSC-focused burden/trend/registration endpoint.
34 / 44	Boennelykke A, Jensen H, Østgård LSG, Falborg AZ, Hansen AT, Christensen KS, Vedsted P. Cancer risk in persons with new-onset anaemia: a population-based cohort study in Denmark. BMC Cancer. 2022 Jul 21;22(1):805. doi: 10.1186/s12885-022-09912-7. PMID: 35864463; PMCID: PMC9306185.	Anaemia/cancer-risk cohort; NMSC was excluded from the main cancer-risk analyses and no eligible NMSC epidemiological result is provided.
35 / 45	Peconi J, O'Neill C, Fegan G, Lanyon K, Driscoll T, Akbari A, Watkins A, Abbott R. Sunproofed study protocol: A mixed-methods scoping study of sun safety policies in primary schools in Wales. PLoS One. 2022 May 11;17(5):e0268141. doi: 10.1371/journal.pone.0268141. PMID: 35544544; PMCID: PMC9094553.	Study protocol only; no outcome data available.
36 / 48	Lecaros-Astorga DA, Molina-Guarneros JA, Rodríguez-Jiménez P, Martin-Arias LH, Sainz-Gil M. Hydrochlorothiazide use and risk of non-melanoma skin cancer in Spain: A case/non-case study. Int J Clin Pharmacol Ther. 2021 Apr;59(4):280-288. doi: 10.5414/CP203769. PMID: 33355085.	Pharmacovigilance disproportionality study; reports drug-safety reporting odds ratios rather than population incidence/prevalence/trends or registry burden.
37 / 49	Keurentjes AJ, Kezic S, Rustemeyer T, Hulshof CTJ, van der Molen HF. Protection Against Solar Ultraviolet Radiation in Outdoor Construction Workers: Study Protocol for a Non-randomized Controlled Intervention Study. Front Public Health. 2021 Mar 4;9:602933. doi: 10.3389/fpubh.2021.602933. PMID: 33748058; PMCID: PMC7969508.	Study protocol only; no outcome data available.

This consolidated exclusion list includes reports examined during supplementary revision checks completed on 30 August 2026. The publication-date eligibility cutoff remained 31 March 2026. Candidate identifiers are screening identifiers, not reference numbers in the manuscript.

PRISMA exclusion-category reconciliation: wrong condition/no separate NMSC outcome (n = 17); secondary etiologic/safety outcome (n = 10); ineligible article type/design or no outcomes (n = 8); outside search period (n = 2). Total full-text exclusions: n = 37.
